# Supplementary material for: Energy landscape reshaped by strain-specific mutations underlies epistasis in NS1 evolution of influenza A virus
Source: Nat Commun. 2022 Oct 1;13:5775. doi: 10.1038/s41467-022-33554-9 (PMC9526705; doi:10.1038/s41467-022-33554-9)
Supplement: Supplementary file 1 — Supplementary Information [file 41467_2022_33554_MOESM1_ESM.pdf]

Supplementary Information for

Energy landscape reshaped by strain-specific mutations underlies epistasis in NS1 evolution of influenza A virus

Iktae Kim[1], Alyssa Dubrow[1], Bryan Zuniga[1], Baoyu Zhao[1], Noah Sherer[1], Abhishek Bastiray[1], Pingwei Li[1], Jae-Hyun Cho[1,*]

[1]Department of Biochemistry and Biophysics, Texas A&M University, College Station, TX77843, USA

*Corresponding author. Email: jaehyun.cho@agnet.tamu.edu

Supplementary Table 1. X-ray diffraction and refinement statistics of the complexes of VN NS^{ED} and $p85\beta^{iSH2}$.

	**VN NS1-ED W187A:p85β complex** PDB ID: 7RCH
**Data collection***	
Space group	$P2_1$
Cell dimensions	
a, *b*, *c* (Å)	61.44, 92.84, 67.12
α, β, γ (°)	90.00, 105.03, 90.00
R_{merge}	0.169 (0.720)
R_{pim}	0.106 (0.442)
$I / \sigma I$	4.0 (1.3)
Completeness (%)	97.8 (96.8)
Redundancy	3.5 (3.6)
**Refinement**	
Resolution (Å)	3.1 (3.31 to 3.10)**
No. Reflections	13026
R_{work}/R_{free}	0.243/0.276
No. atoms	
Protein	4204
Water	0
B-factors	
Overall	74.29
R.m.s. deviations	
Bond lengths (Å)	0.002
Bond angles (°)	0.447

* The numbers in parentheses refer to the highest resolution shell.
** A single crystal was used to collect each of the dataset.

Supplementary Table 2. Chemical shifts of 1918 NS1 methyl atoms.

Residues	Atom name	^{13}C	^{1}H	Residues	Atom name	^{13}C	^{1}H
A79	CB-HB	19.52	1.530	L144	CD1-HD1	23.68	0.774
T80	CG2-HG2	22.00	1.181	L144	CD2-HD2	26.26	0.562
I81	CD1-HD1	13.77	0.885	I145	CD1-HD1	12.41	0.822
I81	CG2-HG2	17.43	0.974	I145	CG2-HG2	17.52	0.925
A82	CB-HB	19.53	1.372	L146	CD1-HD1	22.39	1.199
V84	CG1-HG1	20.85	0.993	L146	CD2-HD2	26.14	1.001
V84	CG2-HG2	20.31	0.931	L147	CD1-HD1	28.94	0.705
A86	CB-HB	19.50	1.312	L147	CD2-HD2	24.01	0.543
L90	CD1-HD1	25.65	0.566	A149	CB-HB	20.39	0.765
L90	CD2-HD2	23.98	0.543	T151	CG2-HG2	22.97	1.240
T91	CG2-HG2	20.74	0.874	A155	CB-HB	19.31	1.374
M93	CE-HE	18.07	1.945	I156	CD1-HD1	15.40	0.692
T94	CG2-HG2	21.93	1.258	I156	CG2-HG2	16.63	-0.753
L95	CD1-HD1	24.12	0.904	V157	CG1-HG1	22.70	0.829
L95	CD2-HD2	24.07	0.874	V157	CG2-HG2	18.60	0.476
M98	CE-HE	17.00	1.995	I160	CD1-HD1	14.20	0.708
M104	CE-HE	17.65	1.535	I160	CG2-HG2	18.66	0.635
L105	CD1-HD1	24.07	0.920	L163	CD1-HD1	26.17	0.821
L105	CD2-HD2	24.37	0.955	L163	CD2-HD2	N.A.	N.A.
M106	CE-HE	N.A.	N.A.	L166	CD1-HD1	N.A.	N.A.
V111	CG1-HG1	21.40	0.904	L166	CD2-HD2	N.A.	N.A.
V111	CG2-HG2	21.52	0.816	T170	CG2-HG2	21.50	1.310
A112	CB-HB	20.12	1.187	V174	CG1-HG1	24.46	0.929
L115	CD1-HD1	26.82	0.489	V174	CG2-HG2	21.09	0.624
L115	CD2-HD2	22.87	0.273	A177	CB-HB	19.42	1.412
I117	CD1-HD1	16.43	0.840	V178	CG1-HG1	24.03	0.733
I117	CG2-HG2	19.97	0.772	V178	CG2-HG2	21.79	0.467
M119	CE-HE	18.07	1.815	V180	CG1-HG1	22.43	1.005
A122	CB-HB	20.42	1.418	V180	CG2-HG2	21.43	0.813
I123	CD1-HD1	9.19	0.757	L181	CD1-HD1	23.48	0.798
I123	CG2-HG2	16.66	0.804	L181	CD2-HD2	27.32	0.619
M124	CE-HE	17.12	2.044	I182	CD1-HD1	13.51	0.338
I128	CD1-HD1	14.51	0.547	I182	CG2-HG2	16.79	0.726
I128	CG2-HG2	18.68	0.631	L185	CD1-HD1	22.09	0.725
I129	CD1-HD1	14.21	0.740	L185	CD2-HD2	26.45	0.547
I129	CG2-HG2	17.49	0.910	A187	CB-HB	17.89	1.523
L130	CD1-HD1	25.44	0.659	T191	CG2-HG2	21.27	1.160
L130	CD2-HD2	24.21	0.559	V192	CG1-HG1	22.69	0.708
A132	CB-HB	25.47	1.383	V192	CG2-HG2	22.82	0.670
V136	CG1-HG1	20.55	0.398	V194	CG1-HG1	21.99	0.827
V136	CG2-HG2	21.48	0.376	V194	CG2-HG2	20.01	0.791
I137	CD1-HD1	13.64	0.695	T197	CG2-HG2	23.83	1.389
I137	CG2-HG2	16.81	0.744	L198	CD1-HD1	24.85	0.610
L141	CD1-HD1	25.27	0.659	L198	CD2-HD2	23.93	0.463
L141	CD2-HD2	26.56	0.317	A202	CB-HB	20.30	1.017
T143	CG2-HG2	22.35	1.097				

Supplementary Table 3. Chemical shifts of PR8 NS1 methyl atoms.

Residues	Atom name	^{13}C	^{1}H	Residues	Atom name	^{13}C	^{1}H
T80	CG2-HG2	21.38	1.253	L144	CD2-HD2	26.16	0.557
M81	CE-HE	16.81	2.083	I145	CD1-HD1	12.25	0.814
A82	CB-HB	19.39	1.383	I145	CG2-HG2	17.60	0.935
V84	CG1-HG1	20.93	0.958	L146	CD1-HD1	25.84	1.025
V84	CG2-HG2	20.35	0.927	L146	CD2-HD2	22.70	1.166
A86	CB-HB	19.41	1.306	L147	CD1-HD1	28.90	0.696
L90	CD1-HD1	25.54	0.538	L147	CD2-HD2	23.90	0.540
L90	CD2-HD2	23.85	0.502	A149	CB-HB	20.44	0.760
T91	CG2-HG2	20.63	0.782	T151	CG2-HG2	22.95	1.243
M93	CE-HE	17.94	1.957	A155	CB-HB	19.28	1.373
T94	CG2-HG2	21.85	1.249	I156	CD1-HD1	15.40	0.703
L95	CD1-HD1	24.17	0.901	I156	CG2-HG2	16.39	-0.799
L95	CD2-HD2	24.16	0.882	V157	CG1-HG1	22.67	0.829
M98	CE-HE	16.97	2.002	V157	CG2-HG2	18.50	0.470
M104	CE-HE	17.49	1.574	I160	CD1-HD1	14.29	0.702
L105	CD1-HD1	24.06	0.900	I160	CG2-HG2	18.30	0.657
L105	CD2-HD2	24.02	0.854	L163	CD1-HD1	25.82	0.923
I106	CD1-HD1	13.16	0.766	L163	CD2-HD2	23.64	0.919
I106	CG2-HG2	17.79	0.823	L166	CD1-HD1	N.A.	N.A.
V111	CG1-HG1	22.15	0.997	L166	CD2-HD2	N.A.	N.A.
V111	CG2-HG2	22.04	0.961	T170	CG2-HG2	21.31	1.292
A112	CB-HB	19.35	1.125	A171	CB-HB	18.22	1.653
L115	CD1-HD1	26.84	0.524	V174	CG1-HG1	24.18	0.909
L115	CD2-HD2	23.26	0.327	V174	CG2-HG2	21.07	0.641
I117	CD1-HD1	16.60	0.858	A177	CB-HB	19.53	1.416
I117	CG2-HG2	19.61	0.760	V178	CG1-HG1	24.14	0.735
M119	CE-HE	18.00	1.828	V178	CG2-HG2	21.76	0.424
A122	CB-HB	20.57	1.428	V180	CG1-HG1	22.09	1.007
I123	CD1-HD1	9.14	0.750	V180	CG2-HG2	21.55	0.824
I123	CG2-HG2	16.59	0.799	L181	CD1-HD1	23.17	0.799
M124	CE-HE	17.05	2.025	L181	CD2-HD2	27.17	0.630
I128	CD1-HD1	14.36	0.546	I182	CD1-HD1	13.49	0.301
I128	CG2-HG2	18.68	0.631	I182	CG2-HG2	16.77	0.718
I129	CD1-HD1	14.18	0.746	L185	CD1-HD1	22.10	0.724
I129	CG2-HG2	17.55	0.904	L185	CD2-HD2	26.49	0.569
L130	CD1-HD1	25.57	0.647	A187	CB-HB	17.83	1.519
L130	CD2-HD2	24.09	0.549	T191	CG2-HG2	21.11	1.152
A132	CB-HB	25.38	1.374	V192	CG1-HG1	22.64	0.708
V136	CG1-HG1	21.43	0.337	V192	CG2-HG2	22.74	0.650
V136	CG2-HG2	20.44	0.361	V194	CG1-HG1	22.07	0.830
I137	CD1-HD1	13.45	0.691	V194	CG2-HG2	20.31	0.773
I137	CG2-HG2	16.73	0.719	T197	CG2-HG2	23.82	1.406
L141	CD1-HD1	25.18	0.657	L198	CD1-HD1	24.55	0.575
L141	CD2-HD2	26.62	0.333	L198	CD2-HD2	23.96	0.422
T143	CG2-HG2	22.46	1.059	A202	CB-HB	20.55	1.031
L144	CD1-HD1	23.68	0.799				

Supplementary Table 4. Chemical shifts of Ud NS1 methyl atoms.

Residues	Atom name	^{13}C	^{1}H	Residues	Atom name	^{13}C	^{1}H
T84	CG2-HG2	21.59	1.258	I145	CD1-HD1	12.82	0.867
A86	CB-HB	19.43	1.317	I145	CG2-HG2	17.16	0.970
I90	CD1-HD1	9.83	0.436	L146	CD1-HD1	22.89	1.195
I90	CG2-HG2	18.18	0.592	L146	CD2-HD2	26.13	0.995
T91	CG2-HG2	20.73	0.856	L147	CD1-HD1	28.92	0.675
M93	CE-HE	18.37	1.980	L147	CD2-HD2	24.14	0.536
T94	CG2-HG2	21.94	1.273	A149	CB-HB	20.59	0.768
I95	CD1-HD1	12.70	0.871	T151	CG2-HG2	23.11	1.227
I95	CG2-HG2	17.44	0.917	A155	CB-HB	19.34	1.360
L98	CD1-HD1	25.55	0.832	I156	CD1-HD1	15.09	0.648
L98	CD2-HD2	25.56	0.833	I156	CG2-HG2	16.51	-0.803
M104	CE-HE	17.61	1.588	V157	CG1-HG1	22.97	0.814
L105	CD1-HD1	24.53	0.954	V157	CG2-HG2	19.17	0.495
L105	CD2-HD2	23.93	0.908	I160	CD1-HD1	14.56	0.675
M106	CE-HE	N.A.	N.A.	I160	CG2-HG2	18.72	0.641
V111	CG1-HG1	22.38	0.975	L163	CD1-HD1	26.25	0.884
V111	CG2-HG2	22.11	0.942	L163	CD2-HD2	24.12	0.970
L115	CD1-HD1	27.23	0.487	T170	CG2-HG2	21.41	1.311
L115	CD2-HD2	23.42	0.328	I171	CD1-HD1	14.47	1.273
I117	CD1-HD1	16.69	0.880	I171	CG2-HG2	16.74	1.073
I117	CG2-HG2	19.40	0.787	V174	CG1-HG1	24.31	0.844
I119	CD1-HD1	14.36	0.765	V174	CG2-HG2	20.83	0.649
I119	CG2-HG2	17.04	0.759	A177	CB-HB	19.55	1.385
A122	CB-HB	20.74	1.418	I178	CD1-HD1	13.93	0.432
I123	CD1-HD1	9.39	0.745	I178	CG2-HG2	17.06	0.114
I123	CG2-HG2	16.74	0.796	V180	CG1-HG1	22.39	1.008
M124	CE-HE	17.20	2.047	V180	CG2-HG2	21.71	0.854
I128	CD1-HD1	14.19	0.537	L181	CD1-HD1	24.24	0.754
I128	CG2-HG2	19.08	0.633	L181	CD2-HD2	27.91	0.617
M129	CE-HE	16.49	1.933	I182	CD1-HD1	13.58	0.419
L130	CD1-HD1	25.37	0.619	I182	CG2-HG2	16.83	0.760
L130	CD2-HD2	24.10	0.546	L185	CD1-HD1	22.49	0.741
A132	CB-HB	25.45	1.422	L185	CD2-HD2	26.68	0.558
V136	CG1-HG1	21.69	0.271	T191	CG2-HG2	21.20	1.152
V136	CG2-HG2	20.87	0.222	V192	CG1-HG1	22.72	0.726
I137	CD1-HD1	13.53	0.693	V192	CG2-HG2	22.92	0.678
I137	CG2-HG2	16.87	0.715	V194	CG1-HG1	22.21	0.816
L141	CD1-HD1	25.18	0.639	V194	CG2-HG2	20.35	0.791
L141	CD2-HD2	27.15	0.003	T197	CG2-HG2	23.63	1.463
T143	CG2-HG2	22.68	1.071	L198	CD1-HD1	25.19	0.682
L144	CD1-HD1	23.97	0.842	L198	CD2-HD2	23.06	0.293
L144	CD2-HD2	26.19	0.559	A202	CB-HB	20.70	0.986

Supplementary Table 5. Chemical shifts of Ud NS1 methyl atoms.

Residues	Atom name	^{13}C	^{1}H	Residues	Atom name	^{13}C	^{1}H
M84	CE-HE	N.A.	N.A.	I145	CG2-HG2	17.77	0.939
A86	CB-HB	19.47	1.317	L146	CD1-HD1	22.54	1.206
L90	CD1-HD1	26.02	0.576	L146	CD2-HD2	26.25	1.002
L90	CD2-HD2	24.05	0.558	L147	CD1-HD1	28.43	0.710
T91	CG2-HG2	20.93	0.802	L147	CD2-HD2	24.18	0.529
M93	CE-HE	18.07	1.925	A149	CB-HB	20.57	0.757
T94	CG2-HG2	22.01	1.253	T151	CG2-HG2	22.99	1.247
L95	CD1-HD1	24.27	0.913	A155	CB-HB	19.23	1.371
L95	CD2-HD2	24.16	0.886	I156	CD1-HD1	15.46	0.673
M98	CE-HE	17.00	1.996	I156	CG2-HG2	16.74	-0.820
M104	CE-HE	17.66	1.609	V157	CG1-HG1	22.68	0.823
L105	CD1-HD1	24.46	0.964	V157	CG2-HG2	18.82	0.468
L105	CD2-HD2	24.31	0.939	I160	CD1-HD1	14.36	0.673
M106	CE-HE	N.A.	N.A.	I160	CG2-HG2	18.74	0.667
V111	CG1-HG1	21.52	0.925	L163	CD1-HD1	25.85	0.912
V111	CG2-HG2	21.52	0.822	L163	CD2-HD2	23.31	0.870
A112	CB-HB	20.18	1.202	L166	CD1-HD1	N.A.	N.A.
L115	CD1-HD1	27.05	0.544	L166	CD2-HD2	N.A.	N.A.
L115	CD2-HD2	23.15	0.247	T170	CG2-HG2	21.50	1.311
I117	CD1-HD1	15.95	0.861	V174	CG1-HG1	26.53	1.013
I117	CG2-HG2	19.57	0.769	V174	CG2-HG2	21.73	0.774
M119	CE-HE	18.03	1.833	A177	CB-HB	19.36	1.435
A122	CB-HB	20.50	1.419	I178	CD1-HD1	14.56	0.526
I123	CD1-HD1	9.32	0.746	I178	CG2-HG2	17.17	0.141
I123	CG2-HG2	16.76	0.806	V180	CG1-HG1	22.63	1.012
M124	CE-HE	17.12	2.050	V180	CG2-HG2	21.52	0.822
T127	CG2-HG2	21.52	1.065	L181	CD1-HD1	23.42	0.803
I128	CD1-HD1	14.38	0.545	L181	CD2-HD2	27.21	0.604
I128	CG2-HG2	18.91	0.620	I182	CD1-HD1	13.22	0.079
I129	CD1-HD1	14.51	0.738	I182	CG2-HG2	16.70	0.715
I129	CG2-HG2	17.83	0.858	L185	CD1-HD1	22.28	0.732
L130	CD1-HD1	25.06	0.624	L185	CD2-HD2	26.59	0.546
L130	CD2-HD2	24.43	0.550	A187	CB-HB	17.89	1.532
A132	CB-HB	24.12	1.174	T191	CG2-HG2	21.12	1.172
V136	CG1-HG1	21.31	0.322	V192	CG1-HG1	22.82	0.714
V136	CG2-HG2	20.57	0.348	V192	CG2-HG2	22.89	0.720
I137	CD1-HD1	13.71	0.689	V194	CG1-HG1	21.99	0.803
I137	CG2-HG2	16.91	0.702	V194	CG2-HG2	19.02	0.905
L141	CD1-HD1	25.45	0.674	T195	CG2-HG2	22.56	1.570
L141	CD2-HD2	26.47	0.218	T197	CG2-HG2	24.82	1.316
T143	CG2-HG2	22.61	1.094	I198	CD1-HD1	14.32	0.859
L144	CD1-HD1	24.15	0.863	I198	CG2-HG2	17.77	0.811
L144	CD2-HD2	26.39	0.585	A202	CB-HB	21.73	1.013
I145	CD1-HD1	12.68	0.826				

Supplementary Figure 1

a

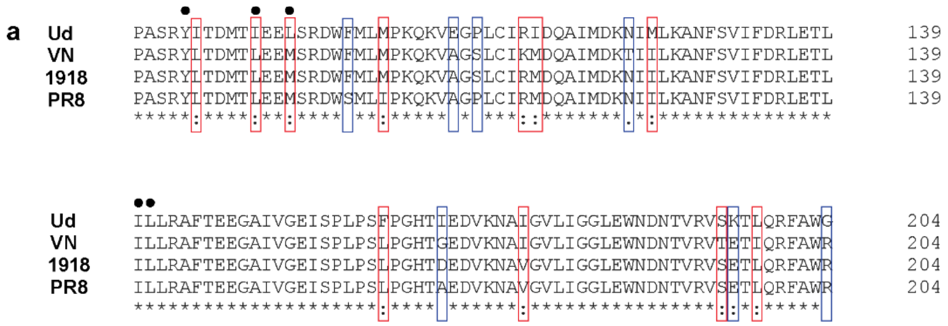

Seq. Identity (%) with respect to	1918	PR8	Ud	VN
1918	100.0			
PR8	96.7	100.0		
Ud	89.0	86.0	100.0	
VN	93.4	91.7	83.0	100.0

b

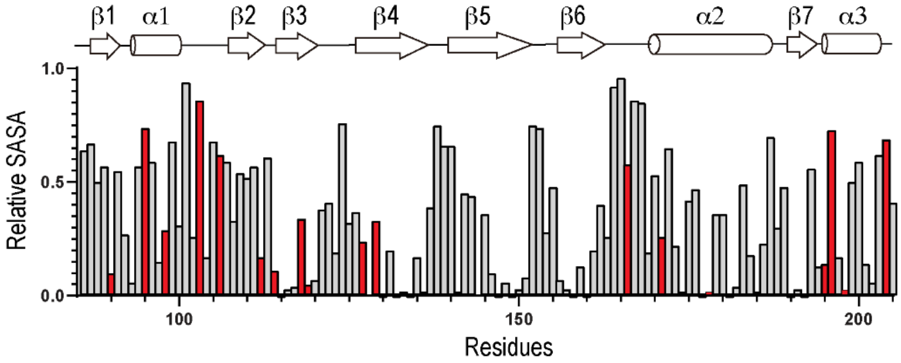

Supplementary Figure 1. **a** Amino acids sequence alignment of NS1 ED proteins (residues 85 – 205). Sequence alignment was performed by the Clustal Omega webserver (https://www.ebi.ac.uk/Tools/msa/clustalo/). Positions for conservative and non-conservative mutations are highlighted by red and blue boxes, respectively. Closed circles represent the positions of core binding residues. Amino acid sequence identities between NS1s of different IAV strains are shown in a matrix format. **b** Relative solvent accessible surface area (SASA) for individual residues of NS1. Positions for strain-specific mutations are highlighted in red. The relative SASA was calculated for 1918 NS1 (PDB ID: 6U28, molecule A) using Pymol.

Supplementary Figure 2

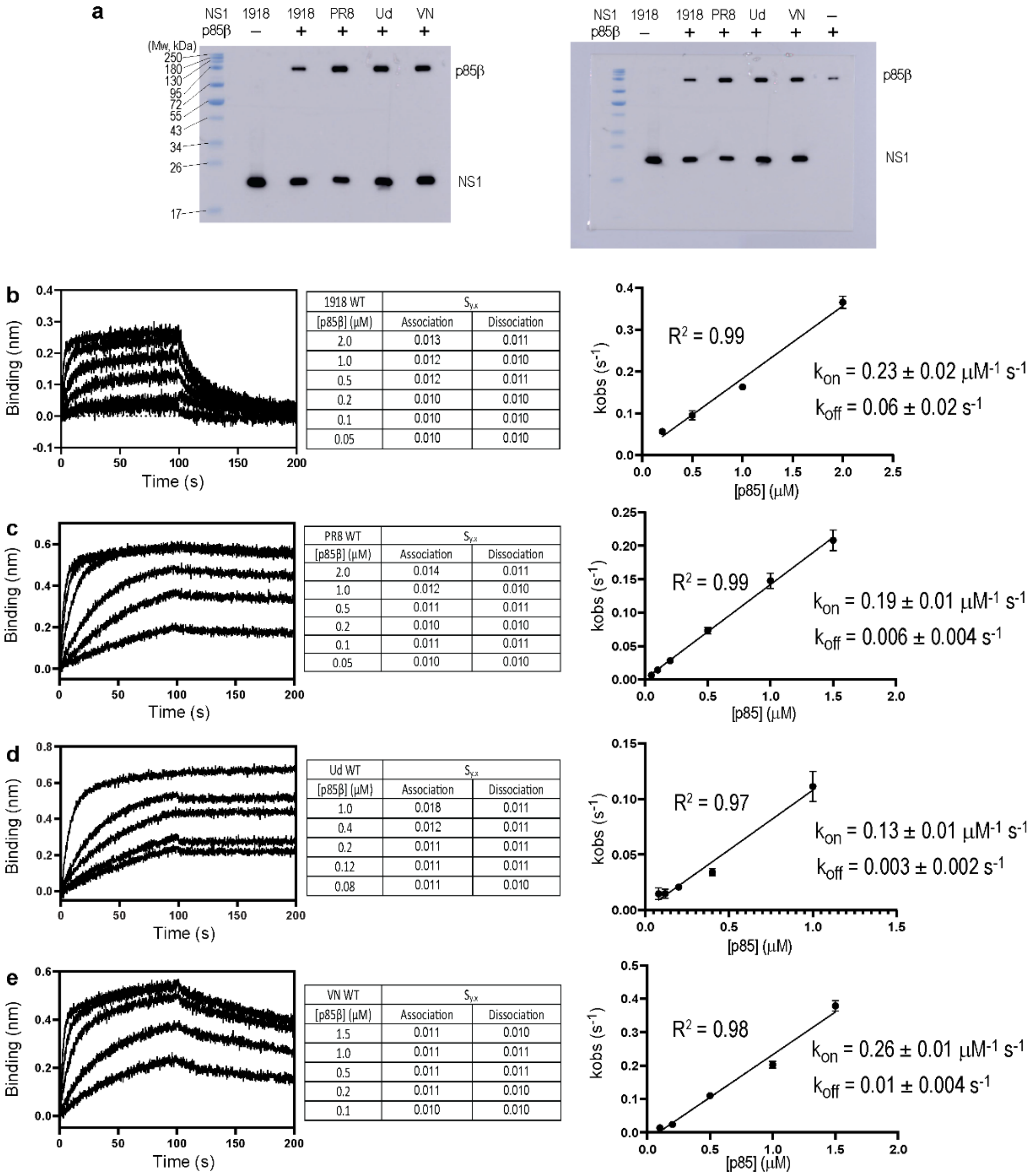

1918 WT	$S_{y.x}$	
[p85β] (μM)	Association	Dissociation
2.0	0.013	0.011
1.0	0.012	0.010
0.5	0.012	0.011
0.2	0.010	0.010
0.1	0.010	0.010
0.05	0.010	0.010

PR8 WT	$S_{y.x}$	
[p85β] (μM)	Association	Dissociation
2.0	0.014	0.011
1.0	0.012	0.010
0.5	0.011	0.011
0.2	0.010	0.010
0.1	0.011	0.011
0.05	0.010	0.010

Ud WT	$S_{y.x}$	
[p85β] (μM)	Association	Dissociation
1.0	0.018	0.011
0.4	0.012	0.011
0.2	0.011	0.011
0.12	0.011	0.011
0.08	0.011	0.010

VN WT	$S_{y.x}$	
[p85β] (μM)	Association	Dissociation
1.5	0.011	0.010
1.0	0.011	0.011
0.5	0.011	0.011
0.2	0.011	0.010
0.1	0.010	0.010

Supplementary Figure 2. Interactions between NS1s and p85β. **a** (left panel) In vitro co-immunoprecipitation of His_6-tagged PI3K by biotin-His_6-tagged NS1s. 3 µg of full-length PI3K (p85β and p110 subunits) was mixed with 3 µg of individual NS1s. The full-length PI3K was purchased from ThermoFisher Scientific (cat. no. A31084). Only p85β has the N-terminal His_6-tag for visualization using the anti-His antibody (Invitrogen Cat No. MA1-21315-HRP, 1:500 dilution, clone: HIS.H8). (right panel) Uncropped gel image. The co-IP and WB were repeated twice, and the results were consistent. Representative BLI sensorgrams and plots of k_{obs} vs [p85] for **b** 1918, **c** Ud, **d** PR8, and **e** VN NS1s. Fit parameters are shown in the plot. Fit parameters and uncertainties are averages and standard deviations of three repeated measurements. $S_{y.x}$ represents the root mean sum-of-squared errors to show the goodness-of-fit of the nonlinear model fit to the BLI data. Source data are provided as a Source Data file.

Supplementary Figure 3

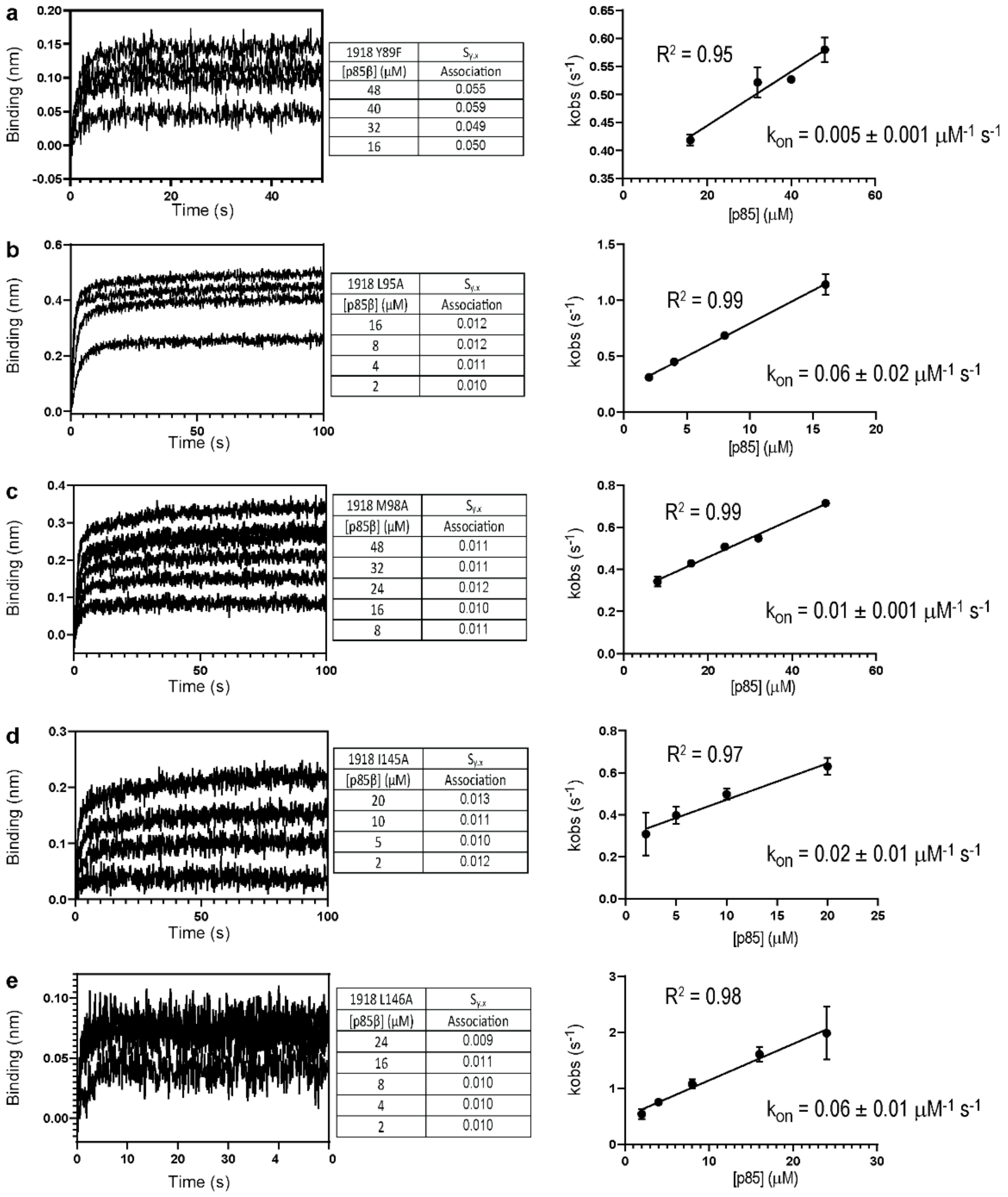

Supplementary Figure 3. Representative BLI sensorgrams (association phases) and plots of k_{obs} vs. [p85] for 1918 NS1 **a** Y89F, **b** L95A, **c** M98A, **d** I145A, and **e** L146A. Fit parameters and uncertainties are averages and standard deviations of three repeated measurements. Closed circles and error bars represent mean and standard deviation of three repeated measurements. $S_{y.x}$ represents the root mean sum-of-squared errors to show the goodness-of-fit of the nonlinear model fit to the BLI data. Source data are provided as a Source Data file.

Supplementary Figure 3 (continued)

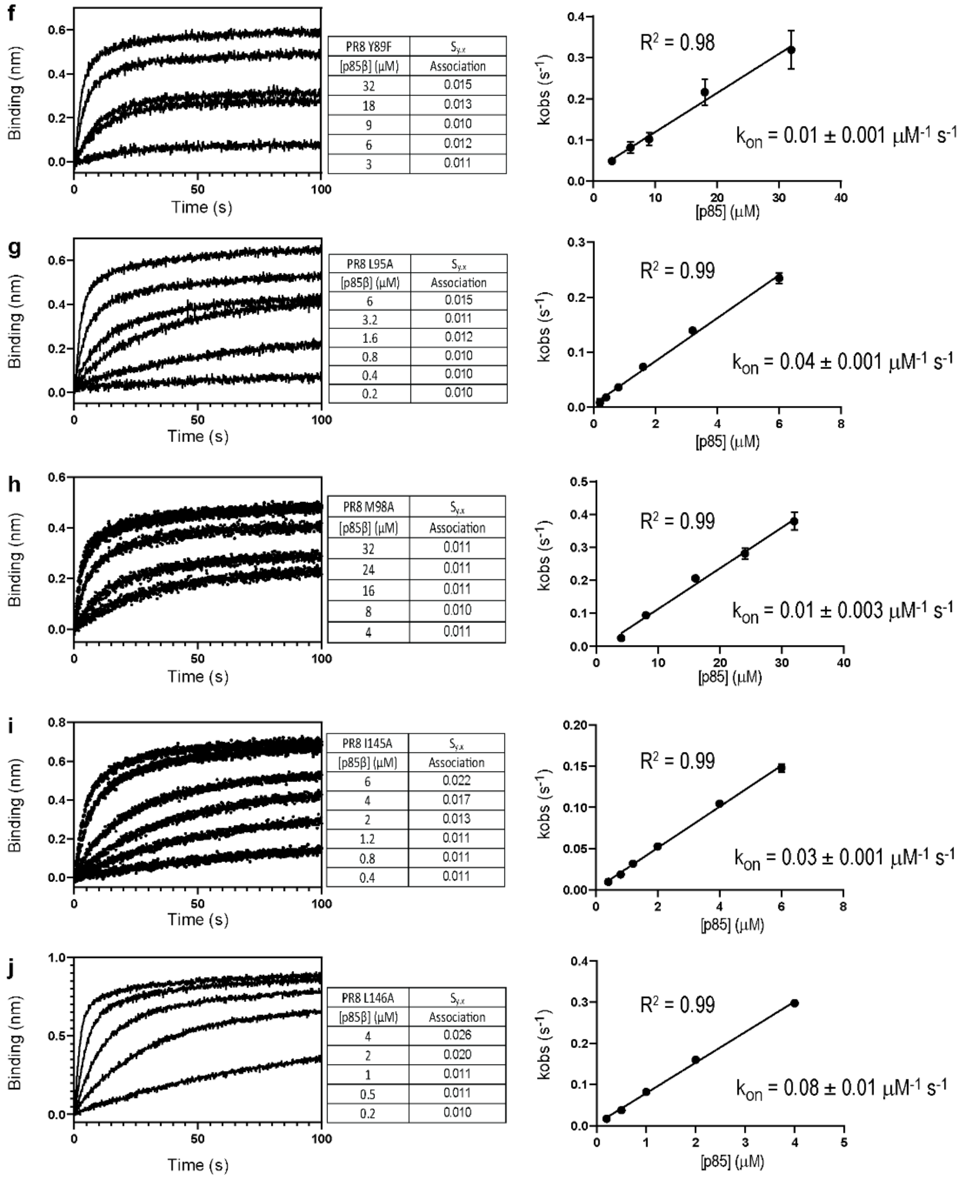

Supplementary Figure 3 (continued). Representative BLI sensorgrams (association phases) and plots of k_{obs} vs. [p85] for PR8 NS1 **f** Y89F, **g** L95A, **h** M98A, **i** I145A, and **j** L146A. Fit parameters and uncertainties are averages and standard deviations of three repeated measurements. Closed circles and error bars represent mean and standard deviation of three repeated measurements. $S_{y.x}$ represents the root mean sum-of-squared errors to show the goodness-of-fit of the nonlinear model fit to the BLI data. Source data are provided as a Source Data file.

Supplementary Figure 3 (continued)

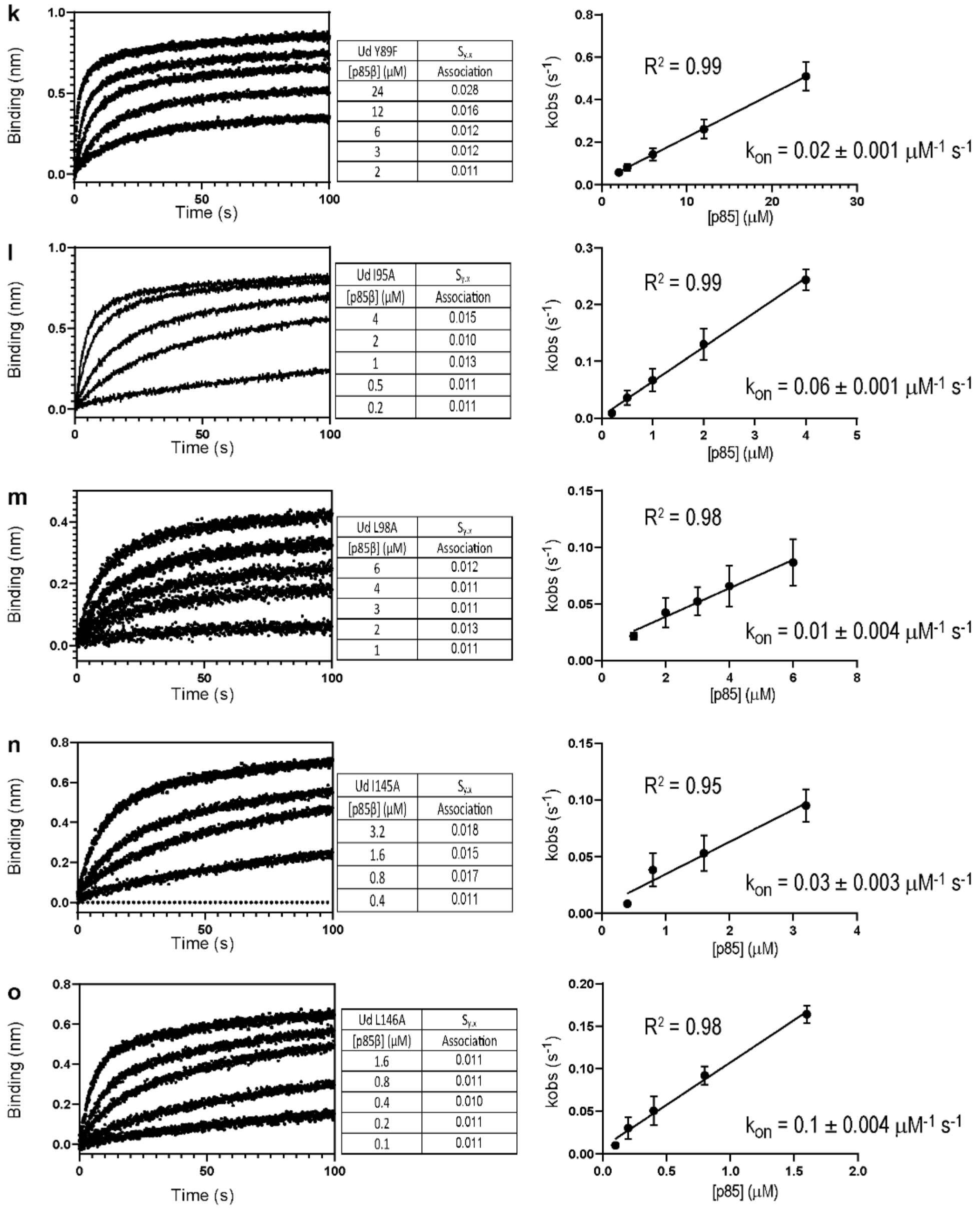

Supplementary Figure 3 (continued). Representative BLI sensorgrams (association phases) and plots of $_{\mathrm{kobs}}$ vs. [p85] for and Ud NS1 **k** Y89F, **l** I95A, **m** L98A, **n** I145A, and **o** L146A. Fit parameters and uncertainties are averages and standard deviations of three repeated measurements. Closed circles and error bars represent mean and standard deviation of three repeated measurements. $S_{y.x}$ represents the root mean sum-of-squared errors to show the goodness-of-fit of the nonlinear model fit to the BLI data. Source data are provided as a Source Data file.

Supplementary Figure 3 (continued)

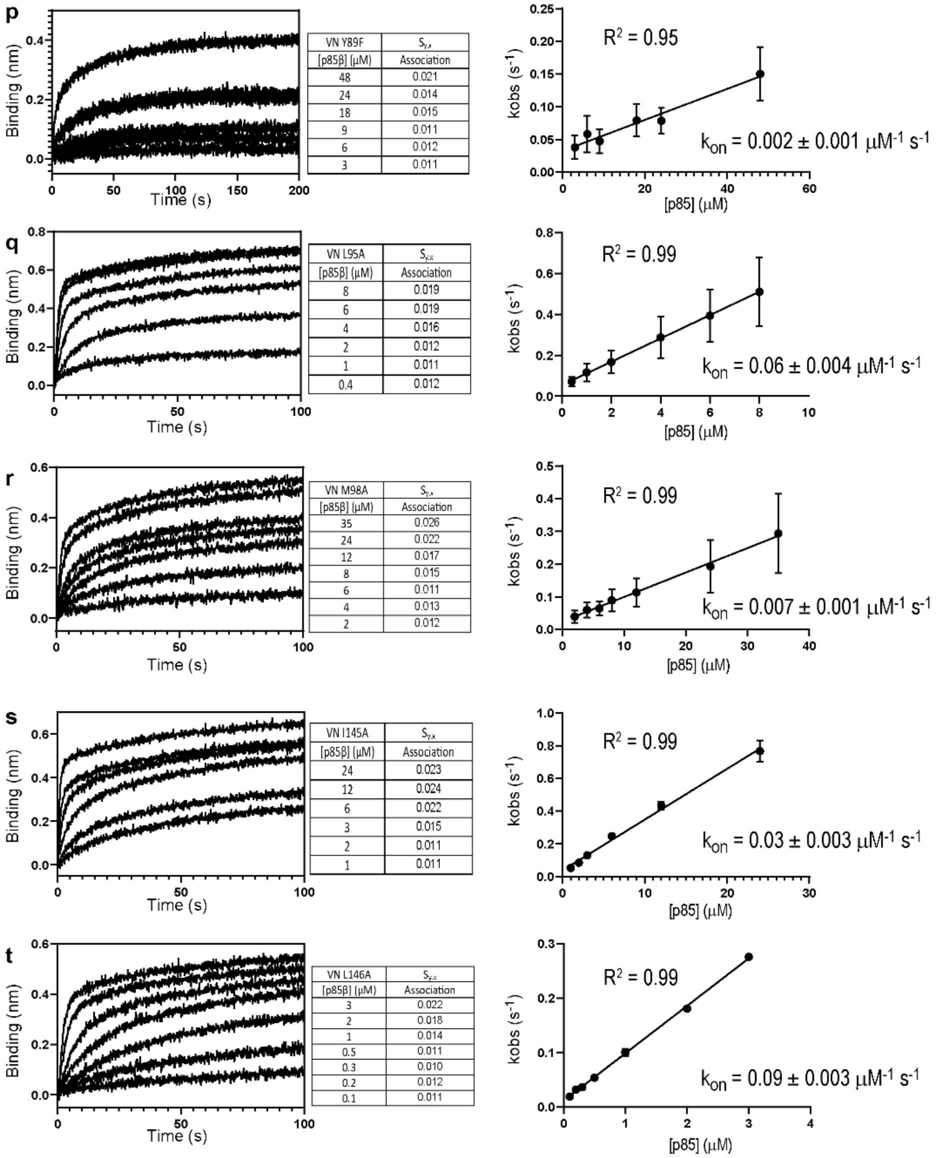

Supplementary Figure 3 (continued). Representative BLI sensorgrams (association phases) and plots of k_{obs} vs. [p85] for VN NS1 **p** Y89F, **q** L95A, **r** M98A, **s** I145A, and **t** L146A. Fit parameters and uncertainties are averages and standard deviations of three repeated measurements. Closed circles and error bars represent mean and standard deviation of three repeated measurements. $S_{y.x}$ represents the root mean sum-of-squared errors to show the goodness-of-fit of the nonlinear model fit to the BLI data. Source data are provided as a Source Data file.

Supplementary Figure 4

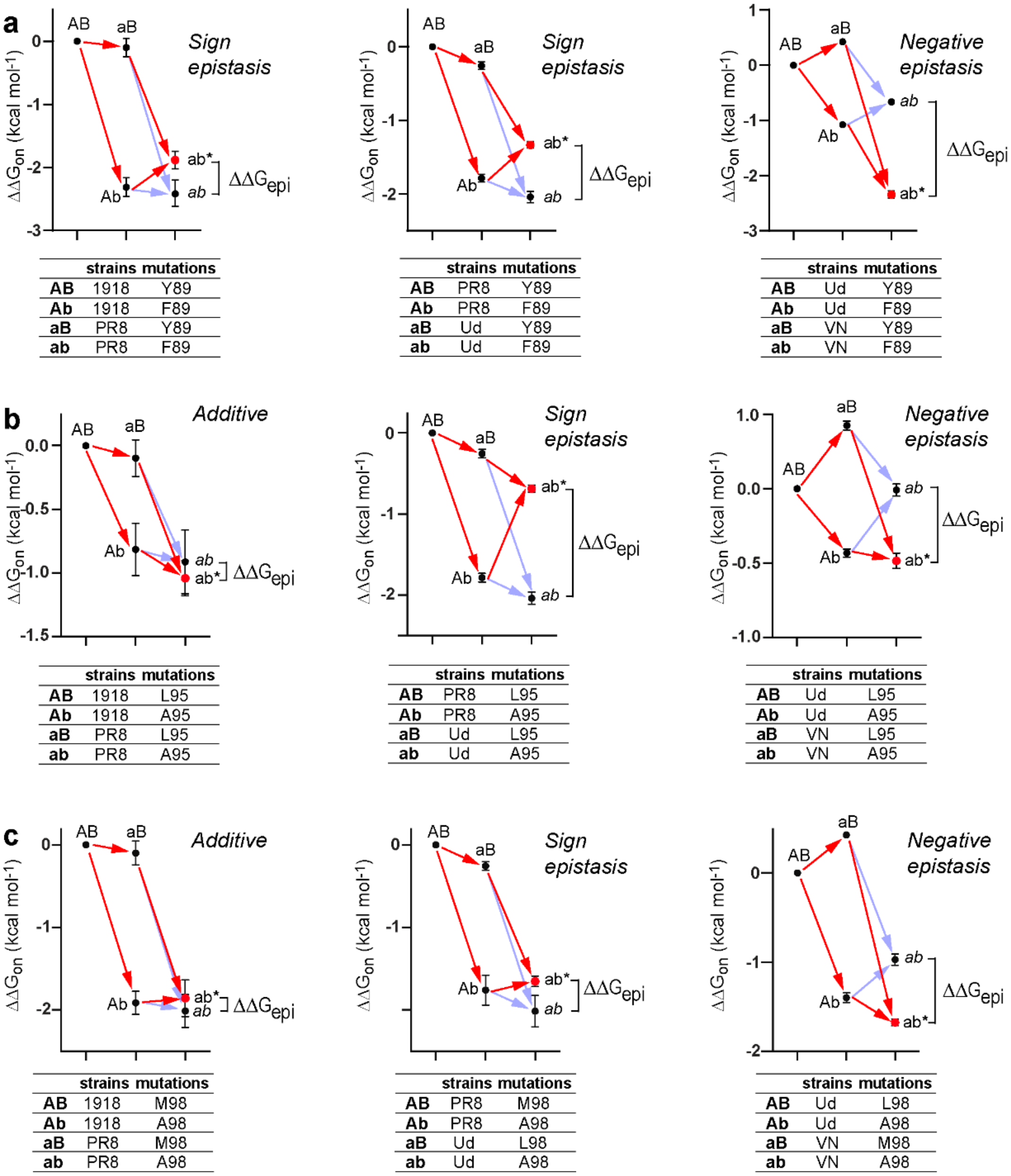

	strains	mutations
**AB**	1918	Y89
**Ab**	1918	F89
**aB**	PR8	Y89
**ab**	PR8	F89

	strains	mutations
**AB**	PR8	Y89
**Ab**	PR8	F89
**aB**	Ud	Y89
**ab**	Ud	F89

	strains	mutations
**AB**	Ud	Y89
**Ab**	Ud	F89
**aB**	VN	Y89
**ab**	VN	F89

	strains	mutations
**AB**	1918	L95
**Ab**	1918	A95
**aB**	PR8	L95
**ab**	PR8	A95

	strains	mutations
**AB**	PR8	L95
**Ab**	PR8	A95
**aB**	Ud	L95
**ab**	Ud	A95

	strains	mutations
**AB**	Ud	L95
**Ab**	Ud	A95
**aB**	VN	L95
**ab**	VN	A95

	strains	mutations
**AB**	1918	M98
**Ab**	1918	A98
**aB**	PR8	M98
**ab**	PR8	A98

	strains	mutations
**AB**	PR8	M98
**Ab**	PR8	A98
**aB**	Ud	L98
**ab**	Ud	A98

	strains	mutations
**AB**	Ud	L98
**Ab**	Ud	A98
**aB**	VN	M98
**ab**	VN	A98

Supplementary Figure 4. Thermodynamic cycle analyses between strain-specific NS1 mutations and core interface mutations on residues **a** 89, **b** 95, **c** 98, **d** 145, and **e** 146. Faint blue arrows represent the expected additive effects of double mutations. Red arrows represent the experimentally measured mutational effects. The difference between ab* and ab defines the pattern of epistasis. $\Delta\Delta G_{epi}$ (ab* – ab) corresponds to the strength of epistatic interactions. Closed symbols represent $\Delta\Delta G_{on}$ values calculated using mean k_{on} values. Error bars represent the propagated standard deviation of three repeated measurements of k_{on} values. Source data are provided as a Source Data file.

Supplementary Figure 4 (continued)

d

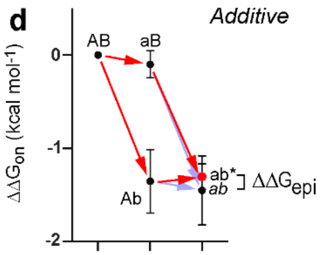

strains	mutations	
**AB**	1918	I145
**Ab**	1918	A145
**aB**	PR8	I145
**ab**	PR8	A145

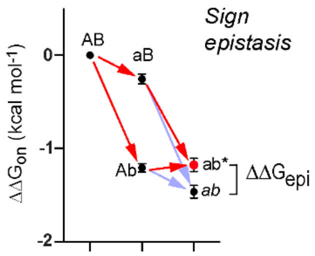

strains	mutations	
**AB**	PR8	I145
**Ab**	PR8	A145
**aB**	Ud	I145
**ab**	Ud	A145

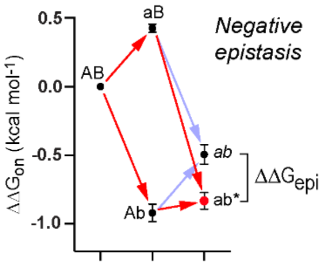

strains	mutations	
**AB**	Ud	I145
**Ab**	Ud	A145
**aB**	VN	I145
**ab**	VN	A145

e

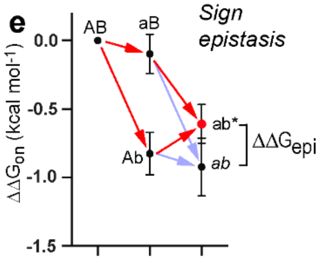

strains	mutations	
**AB**	1918	L146
**Ab**	1918	A146
**aB**	PR8	L146
**ab**	PR8	A146

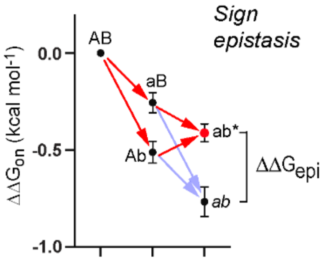

strains	mutations	
**AB**	PR8	L146
**Ab**	PR8	A146
**aB**	Ud	L146
**ab**	Ud	A146

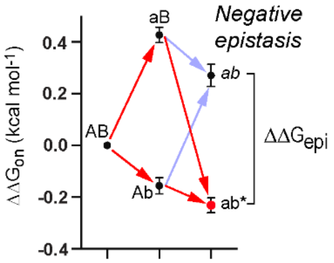

strains	mutations	
**AB**	Ud	L146
**Ab**	Ud	A146
**aB**	VN	L146
**ab**	VN	A146

Supplementary Figure 5

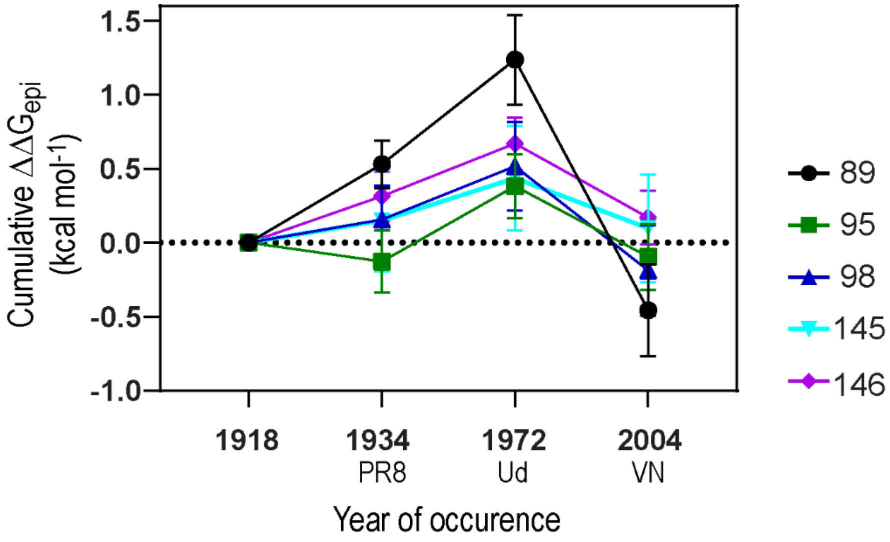

Supplementary Figure 5. Cumulative epistatic interactions with respect to 1918 NS1. Cumulative values were calculated by adding $\Delta\Delta G_{epi}$ values from 1918. For example, cumulative $\Delta\Delta G_{epi}$ of Y89F ($\Delta\Delta G_{epi}^{Y89F}$) from 1918 to 2004 (VN) was calculated as follows; $\Delta\Delta G_{epi}^{Y89F}(2004 – 1918) = \Delta\Delta G_{epi}^{Y89F}(1934 – 1918) + \Delta\Delta G_{epi}^{Y89F}(1972 – 1934) + \Delta\Delta G_{epi}^{Y89F}(2004 – 1972)$. Individual $\Delta\Delta G_{epi}$(A – B) values are defined in Supplementary Figure 4, where A and B correspond to two different years. For example, $\Delta\Delta G_{epi}^{Y89F}$(1934 – 1918), $\Delta\Delta G_{epi}^{Y89F}$(1972 – 1934), and $\Delta\Delta G_{epi}^{Y89F}$(2004 – 1972) correspond to $\Delta\Delta G_{epi}$ defined in the left, middle, and right panels of Fig S4A. Closed symbols represent the $\Delta\Delta G_{epi}$ values calculated using mean k_{on} values. Error bars represent the propagated standard deviation of three repeated measurements of k_{on} values. Source data are provided as a Source Data file.

Supplementary Figure 6

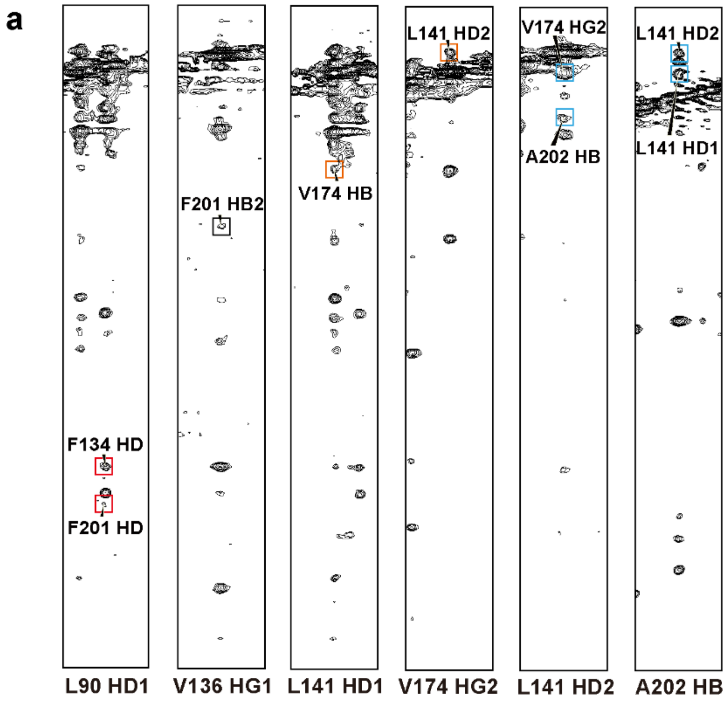

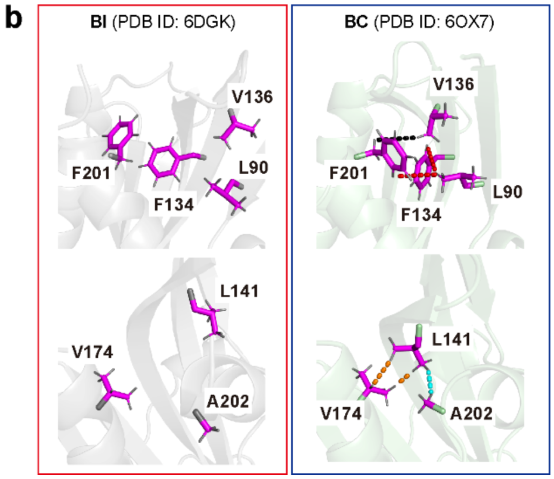

c

Atom	Atom	Distance BI (Å)	Distance BC (Å)
L90 HD	F134 HD	7.0	4.3
L90 HD	F201 HD	9.5	4.5
V136 HG	F201 HB	7.8	3.9
L141 HD	V174 HB	8.6	2.6
L141 HD	V174 HG	7.0	2.7
L141 HD	A202 HB	7.7	2.1

Supplementary Figure 6. a NMR NOESY spectra of free 1918 NS1 and **b** crystal structures corresponding to BI and BC conformers of free 1918 NS1. The identified ^{1}H atom pairs are marked by dotted lines in the BC structures (shown in green cartoon) in the same color as the corresponding NOESY cross-peaks. The same ^{1}H atom pairs are > 5 Å in the BI conformer (gray). **c** Pairwise distances included in this analysis are shown in a table format.

Supplementary Figure 6 (continued)

d

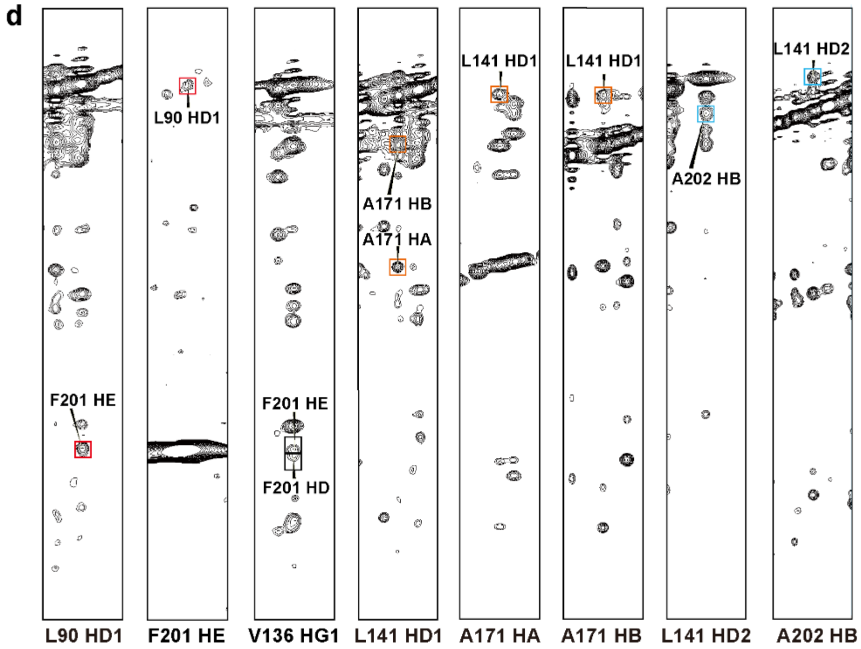

e

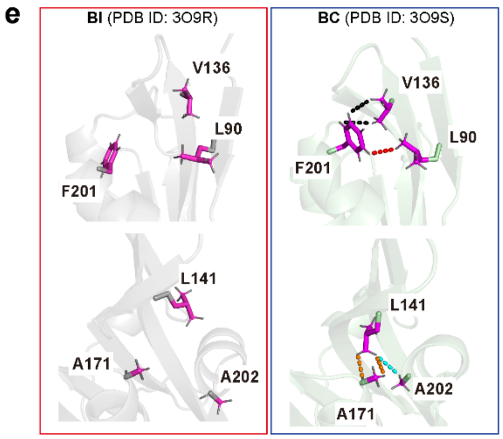

f

Atom	Atom	Distance BI (Å)	Distance BC (Å)
L90 HD	F201 HE	8.9	3.1
V136 HG	F201HD	7.3	3.2
V136 HG	F201 HE	7.8	3.3
L141 HD	A171 HA	8.3	2.5
L141 HD	A171 HB	7.5	3.3
L141 HD	A202 HB	8.5	3.6

Supplementary Figure 6 (continued). d NMR NOESY spectra of free PR8 NS1 and **e** crystal structures corresponding to BI and BC conformers of free PR8 NS1. The identified ^{1}H atom pairs are marked by dotted lines in the BC structures (shown in green cartoon) in the same color as the corresponding NOESY cross-peaks. The same ^{1}H atom pairs are > 5 Å in the BI conformer (gray). **f** Pairwise distances included in this analysis are shown in a table format.

Supplementary Figure 6 (continued)

g

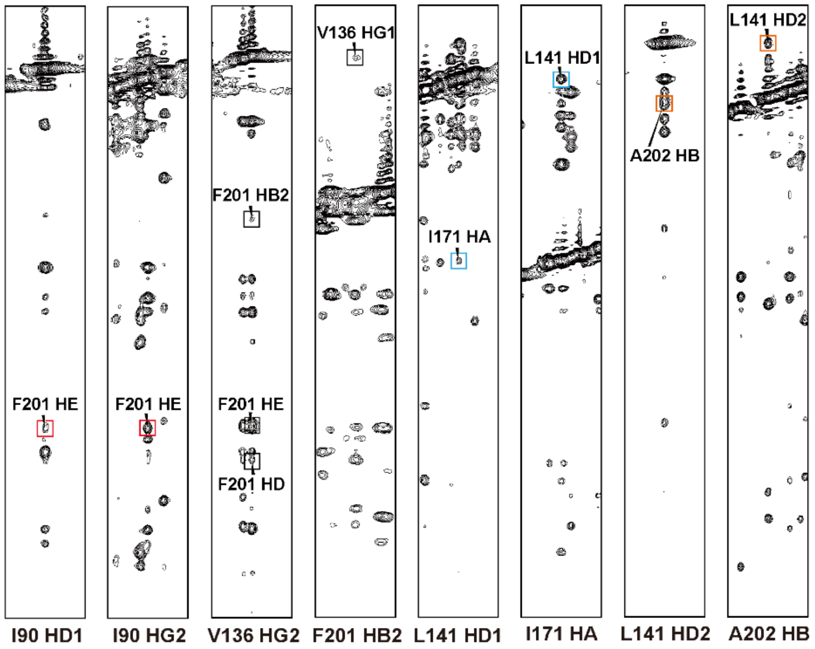

h

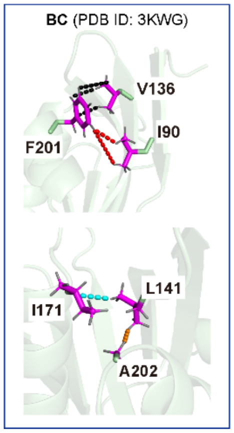

i

Atom	Atom	Distance BI (Å)	Distance BC (Å)
I90 HD1	F201 HE	NA	3.8
I90 HG2	F201 HE	NA	3.4
V136 HG	F201 HB	NA	2.6
V136 HG	F201 HD	NA	2.8
V136 HG	F201 HE	NA	3.4
L141 HD	I171 HA	NA	2.8
L141 HD	A202 HB	NA	2.7

NA: Not available.

Supplementary Figure 6 (continued). g NMR NOESY spectra of free Ud NS1 and the **h** crystal structure corresponding to the BC conformer. The identified ^{1}H atom pairs are marked by dotted lines in the BC structures (shown in green cartoon) in the same color as the corresponding NOESY cross-peaks. There is no structure corresponding to the BI conformer of Ud NS1 in PDB. **i** Pairwise distances included in this analysis are shown in a table format.

Supplementary Figure 6 (continued)

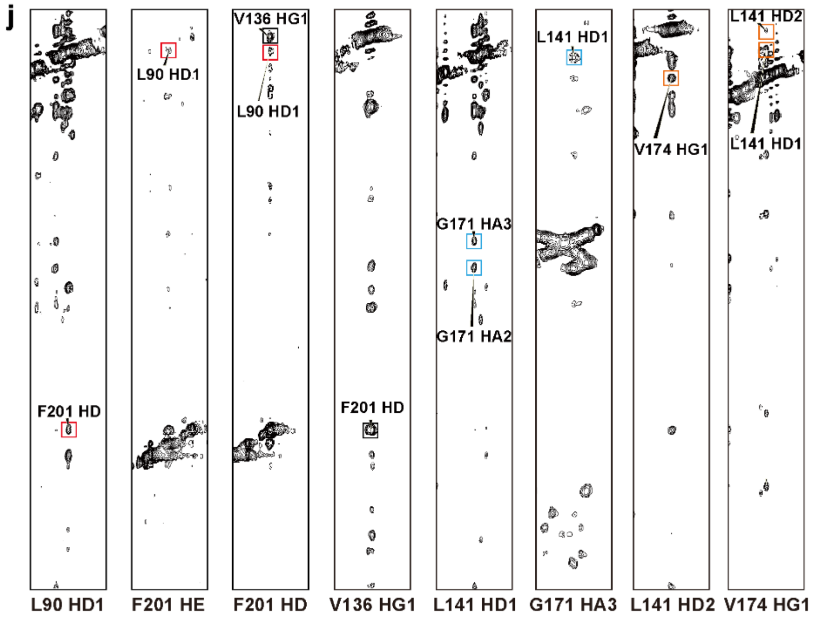

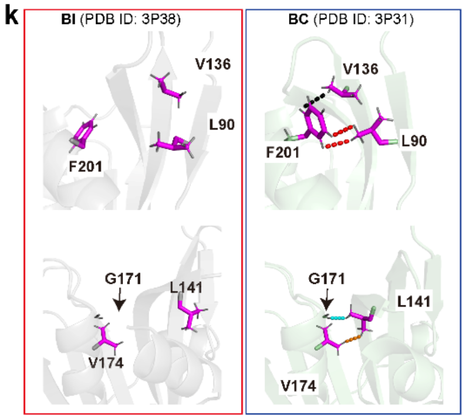

l

Atom	Atom	Distance BI (Å)	Distance BC (Å)
L90 HD	F201 HD	7.7	2.8
L90 HD	F201 HE	12.8	3.1
V136 HG	F201 HD	8.6	3.2
L141 HD	G171 HA	9.3	2.9
L141 HD	V174 HG	7.2	2.4

Supplementary Figure 6 (continued). j NMR NOESY spectra of free VN NS1 and **k** crystal structures corresponding to BI and BC conformers of free VN NS1. The identified ^{1}H atom pairs are marked by dotted lines in the BC structures (shown in green cartoon) in the same color as the corresponding NOESY cross-peaks. The same ^{1}H atom pairs are > 5 Å in the BI conformer (gray). **l** Pairwise distances included in this analysis are shown in a table format.

Supplementary Figure 7

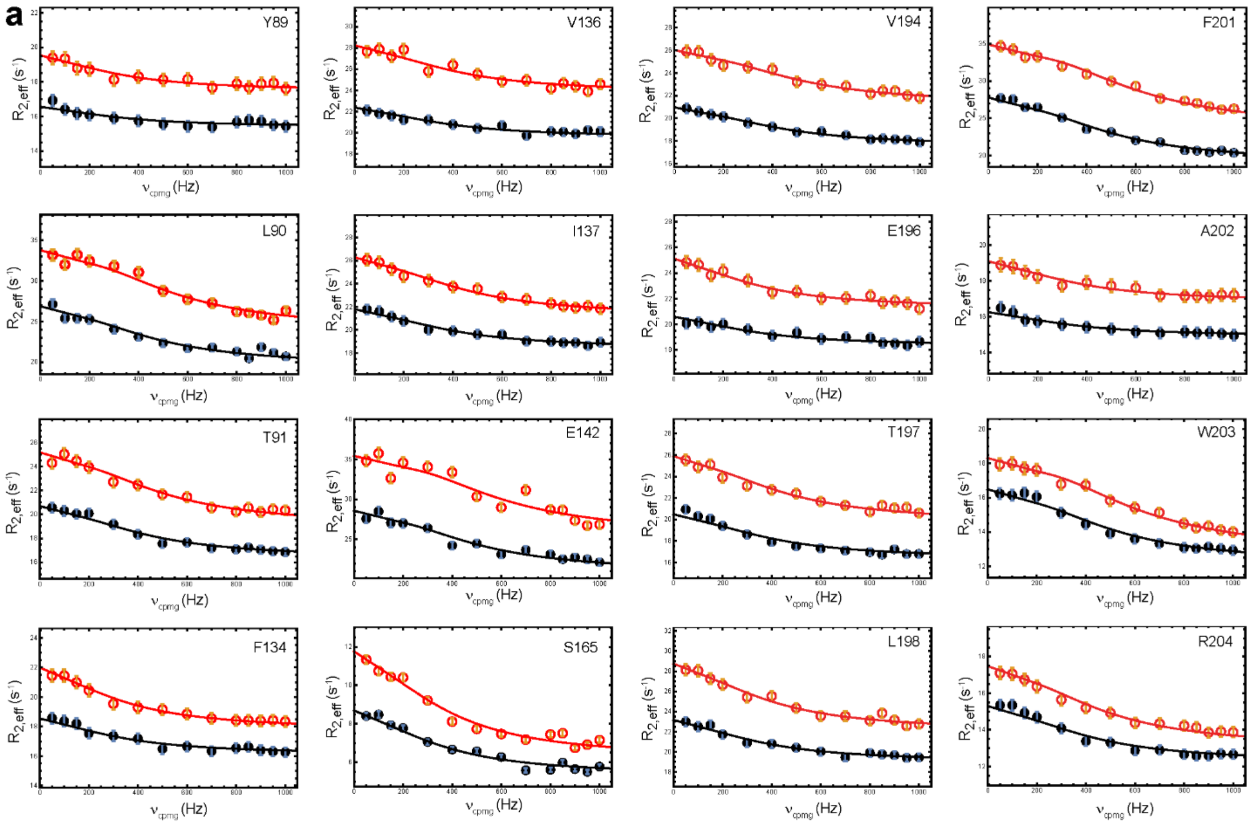

Supplementary Figure 7. a NMR ^{15}N CPMG-RD data of free PR8 NS1. Data at both 600- (black circles) and 800-MHz (red circles) were globally fit (solid lines) using the Carver-Richards equation with an assumption of a simple two-state exchange model. All residues were fit with a globally constrained k_{ex} value (2200 ± 930 s^{-1}). The population of the minor species was derived from slow-exchanging residues (residues 90, 134, 137, 197, and 201). Circles and error bars represent the best fit values and the standard error of the fitted parameter, respectively. Source data are provided as a Source Data file.

Supplementary Figure 7 (continued)

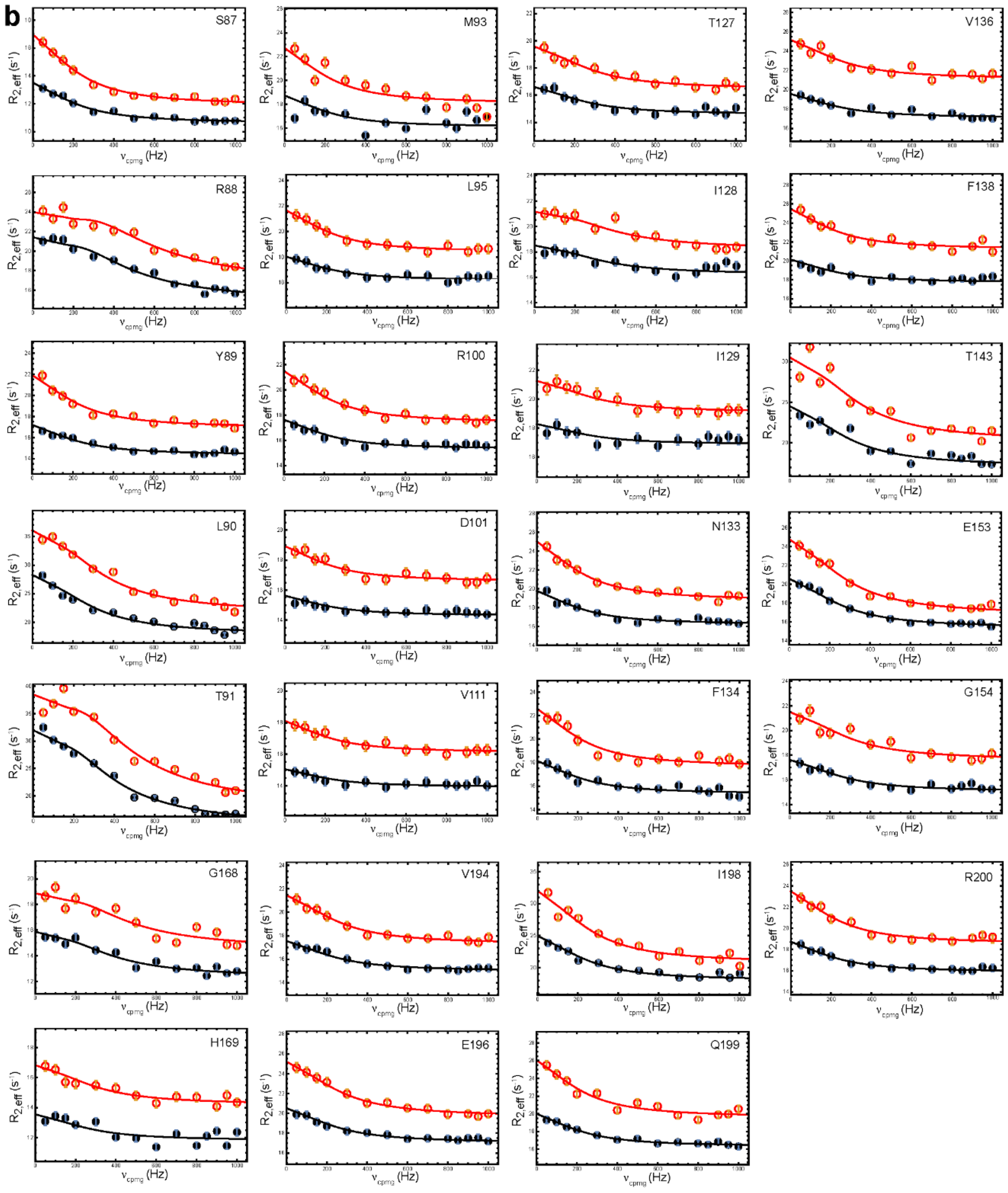

Supplementary Figure 7 (continued). b NMR ^{15}N CPMG-RD data of free VN NS1. Data at both 600- (black circles) and 800-MHz (red circles) were globally fit (solid lines) using the Carver-Richards equation with an assumption of a simple two-state exchange model. All residues were fit with a globally constrained k_{ex} value (1500 ± 400 s^{-1}). The population of the minor species was derived from slow-exchanging residues (residues 88, 90, 91,127, and 153). Circles rand error bars represent the best fit values and standard error of the fitted parameter, respectively. Source data are provided as a Source Data file.

Supplementary Figure 8

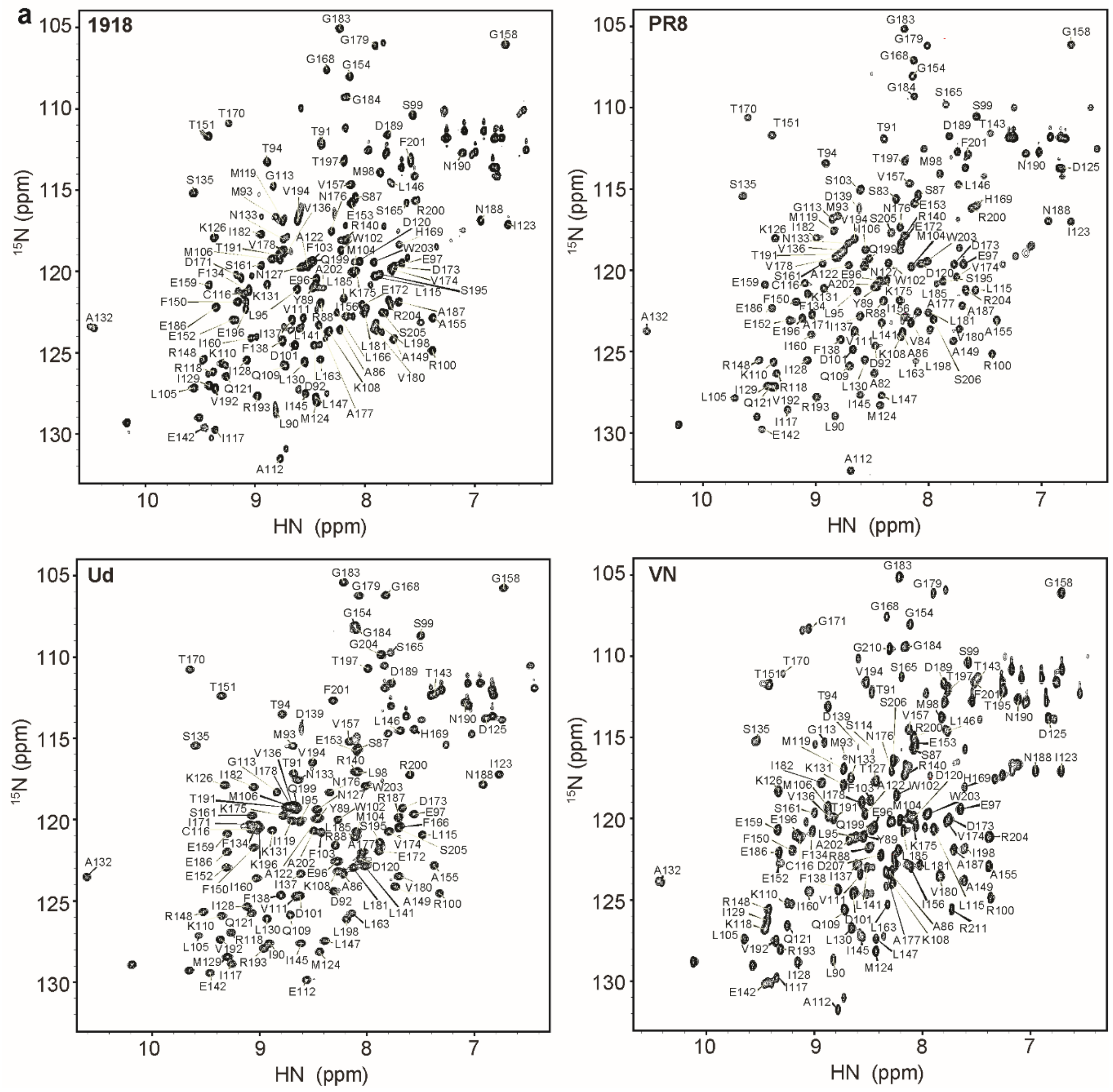

Supplementary Figure 8. a ^{1}H-^{15}N HSQC spectra of free NS1 proteins. The assignments of backbone resonances are available from the BMRB: 1918 (BMRB accession number: 12032), PR8 (BMRB accession number: 51404), Ud (BMRB accession number: 16376), and VN (BMRB accession number: 51403).

Supplementary Figure 8 (continued)

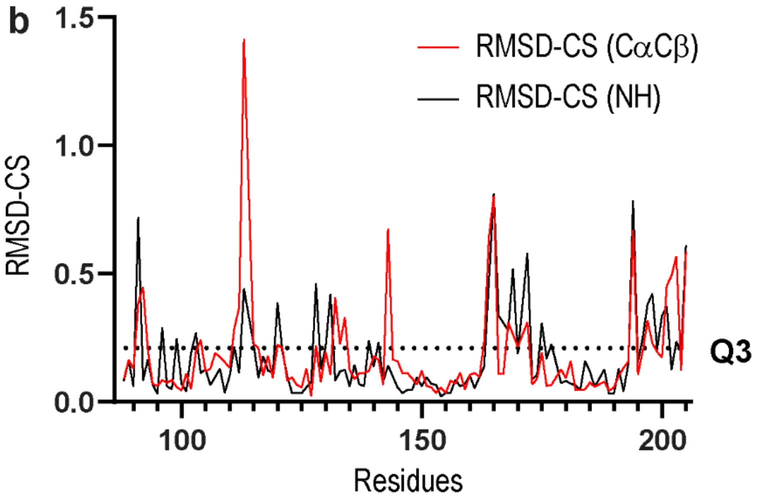

Supplementary Figure 8 (continued). b The RMSD-CS plots of combined $^{13}C_{\alpha}$ and $^{13}C_{\beta}$ resonances (red line) and backbone amide (^{1}H and ^{15}NH) resonances (black line) of four NS1s (n = 4). Q3 values for RMSD-CS ($^{13}C_{\alpha}$ and $^{13}C_{\beta}$) and RMSD-CS (^{1}H and ^{15}NH) are 0.21 and 0.20, respectively. Source data are provided as a Source Data file.

Supplementary Figure 9

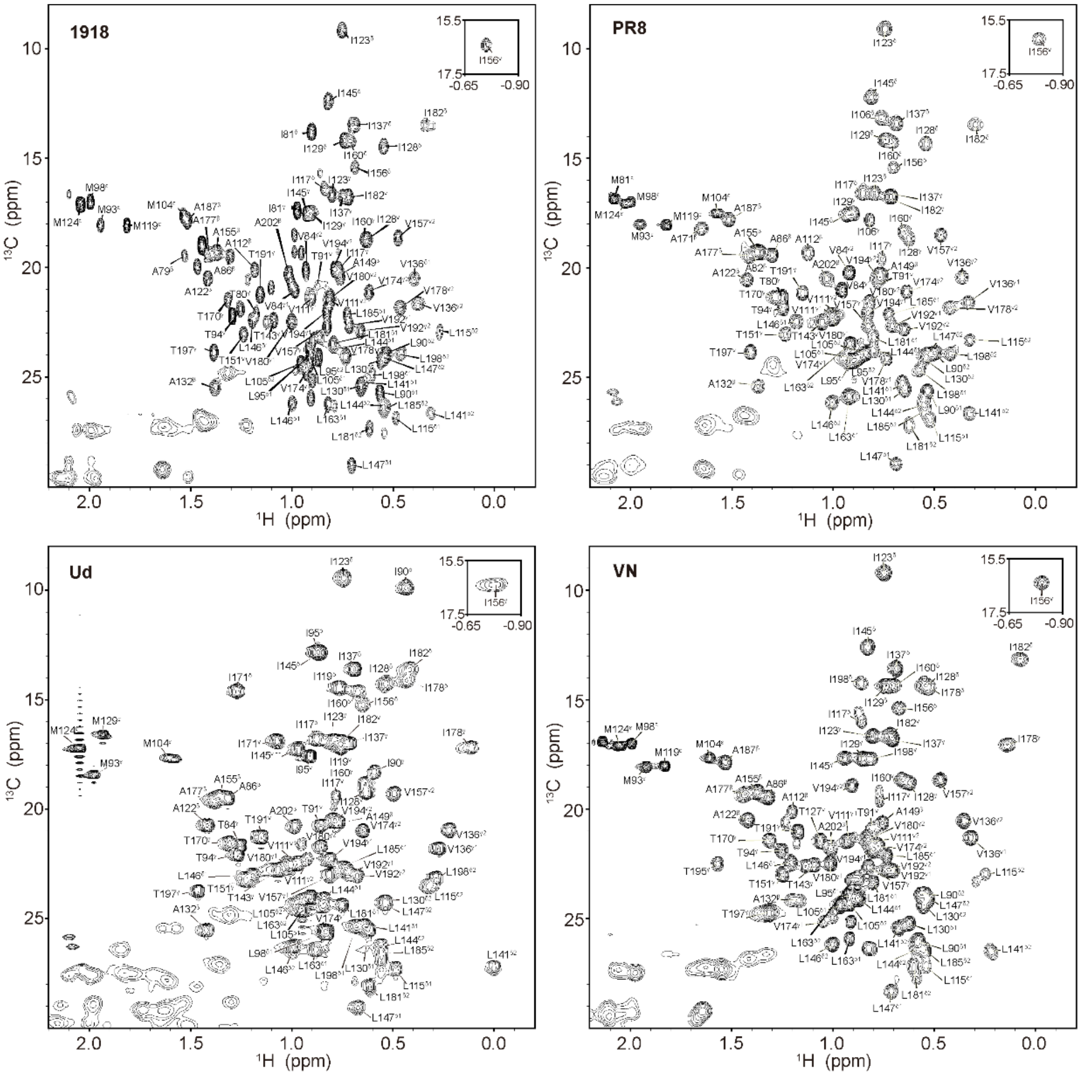

Supplementary Figure 9. ^{1}H-^{13}C HSQC spectra for methyl resonances of free NS1 proteins. The assignments of methyl resonances are shown in Supplementary Tables 2-5.

Supplementary Figure 10

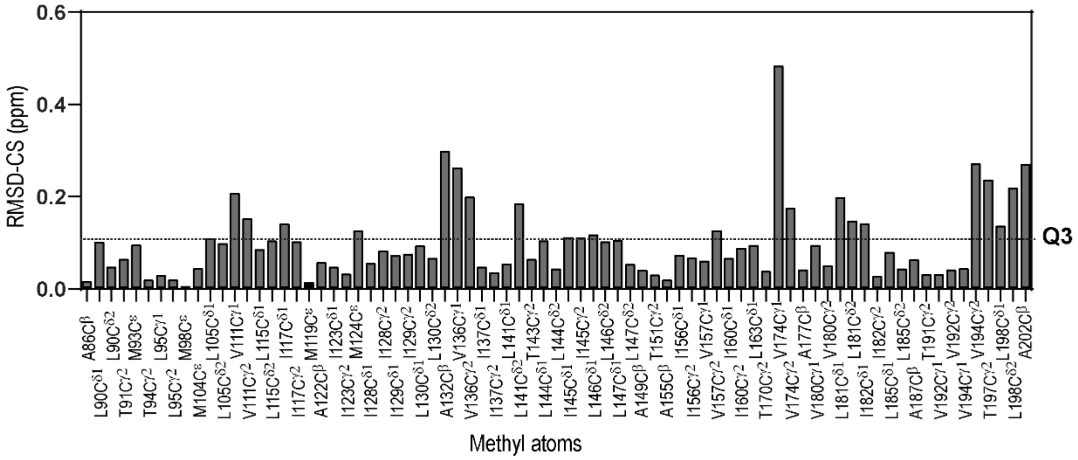

Supplementary Figure 10. RMSD of methyl resonances (^{1}H and ^{13}C) of four NS1s (n = 4). The dotted line represents Q3 of RMSD values. Source data are provided as a Source Data file.

Supplementary Figure 11

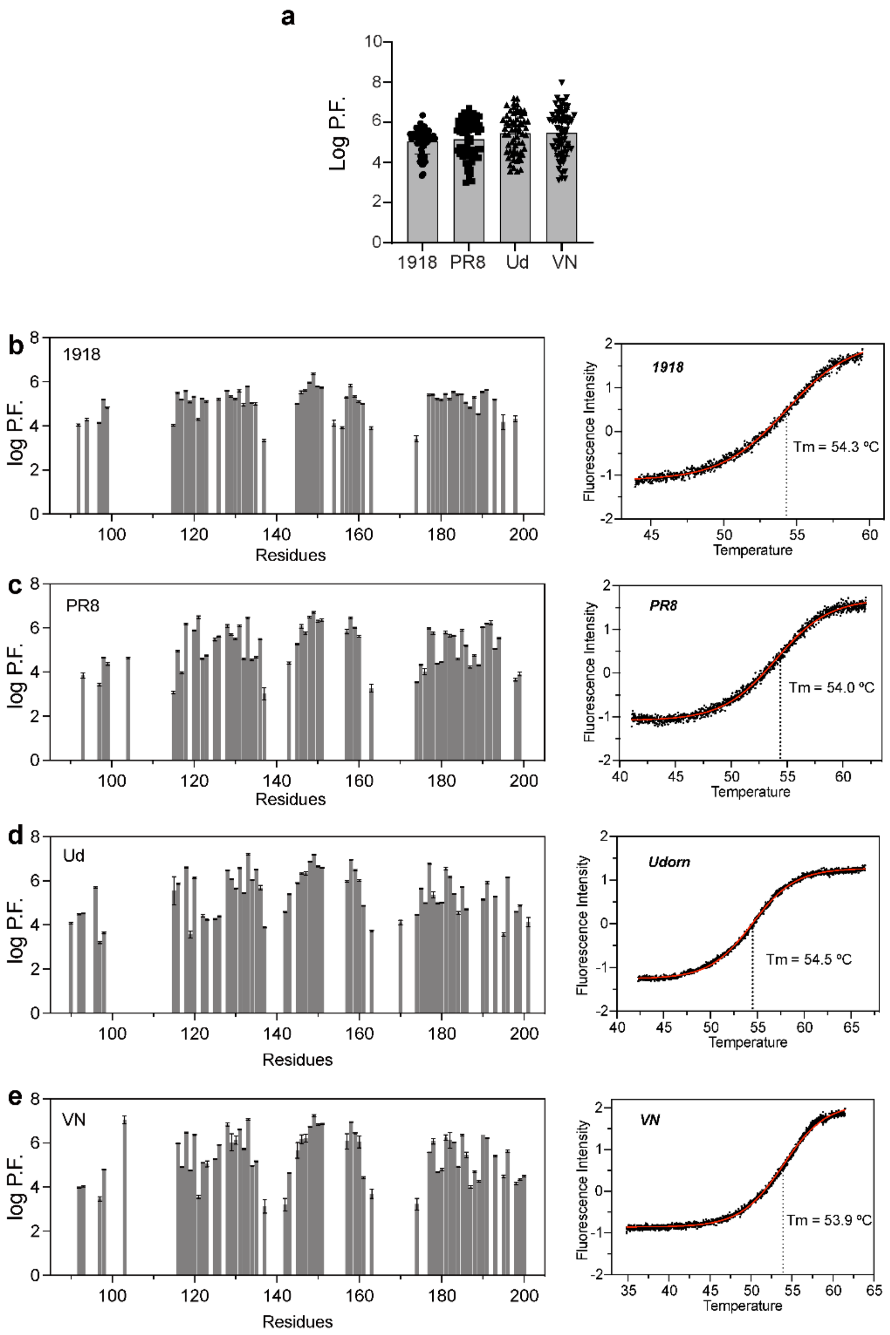

Supplementary Figure 11. a Average protection factors (P.F.) of backbone amide proton exchange of NS1s in D_2O. Bars represent the mean exchange rates of all measured residues in a protein. Filled symbols represent the individual data points. Log (PF) of individual residues (left panel) and protein melting curves (right panel) of **b** 1918, **c** PR8, **d** Ud, and **e** VN NS1s. **b – e** Bars and whiskers represent the best fit values and standard error of the fitted parameter, respectively. Source data are provided as a Source Data file.

Supplementary Figure 12

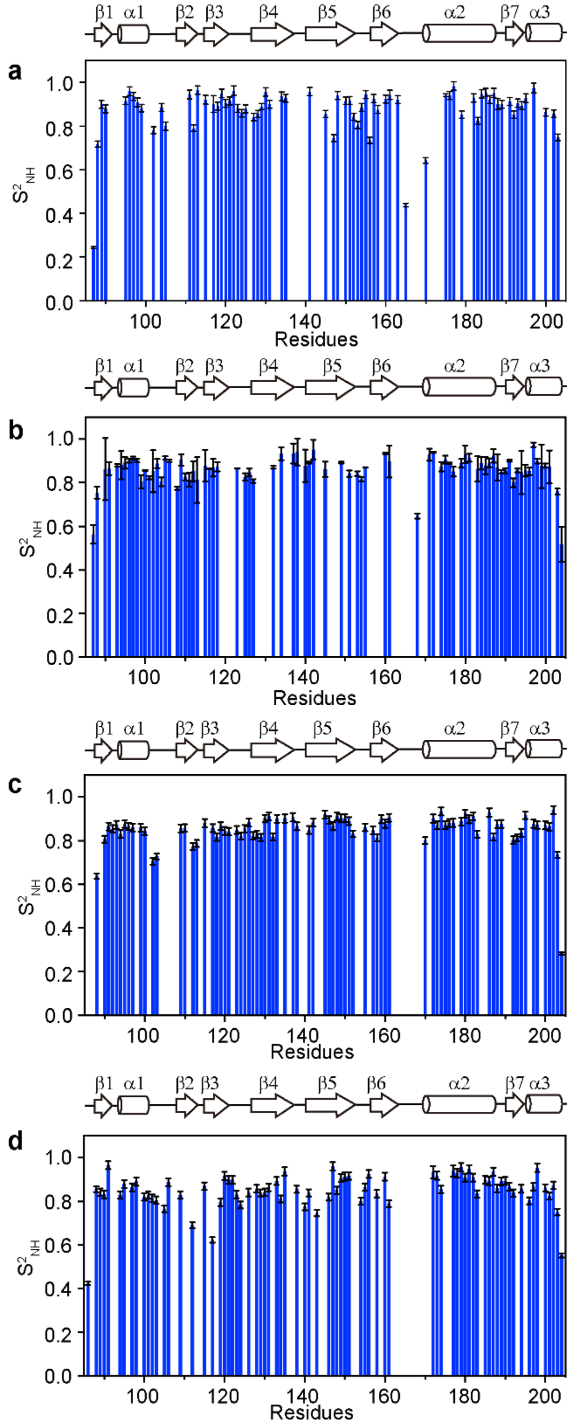

Supplementary Figure 12. Order parameters for backbone ^{15}N-H vectors (S^2_{NH}) of **a** 1918, **b** PR8, **c** Ud, and **d** VN NS1s in the free state. Bars and whiskers represent the best fit values and standard errors of the fitted parameter, respectively. Source data are provided as a Source Data file.

Supplementary Figure 13

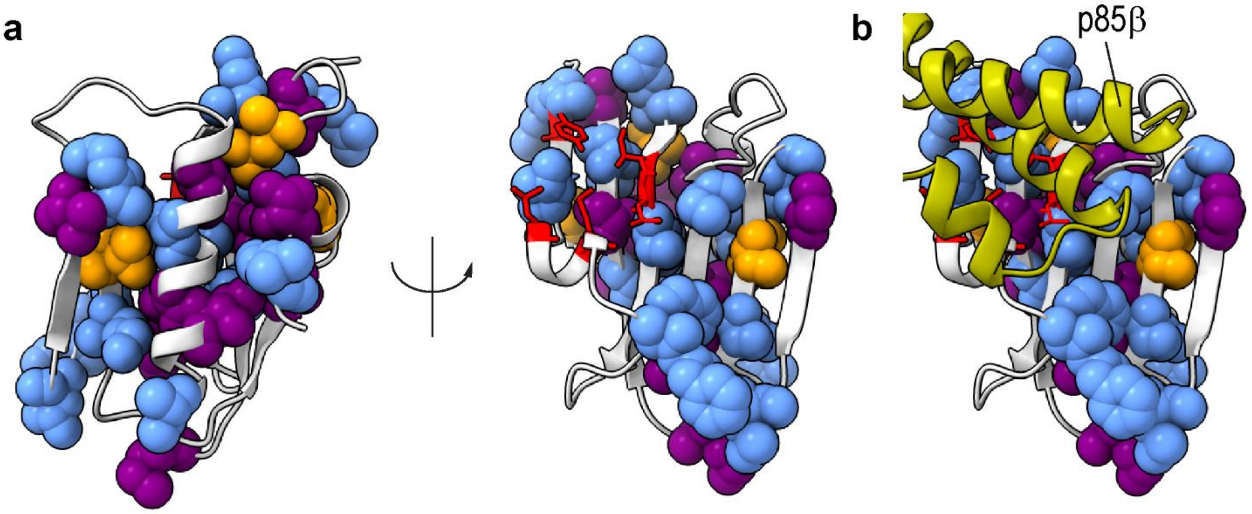

Supplementary Figure 13. Structure of NS1 (1918 NS1, PDB ID: 6U28). **a** Residues whose backbone is dynamically variable across NS1s (i.e., S^2_{NH} > Q3) are shown in blue spheres. Residues whose side-chain is conformationally variable across NS1s (i.e., RMSD-CS,$^{13}CH_3$ > Q3) are shown in purple spheres. Residues with both dynamically variable backbone and conformationally variable side-chain are shown in orange. Core interface residues are shown in red sticks. **b** The bound p85β is shown to display the binding interface on NS1.

Supplementary Figure 14

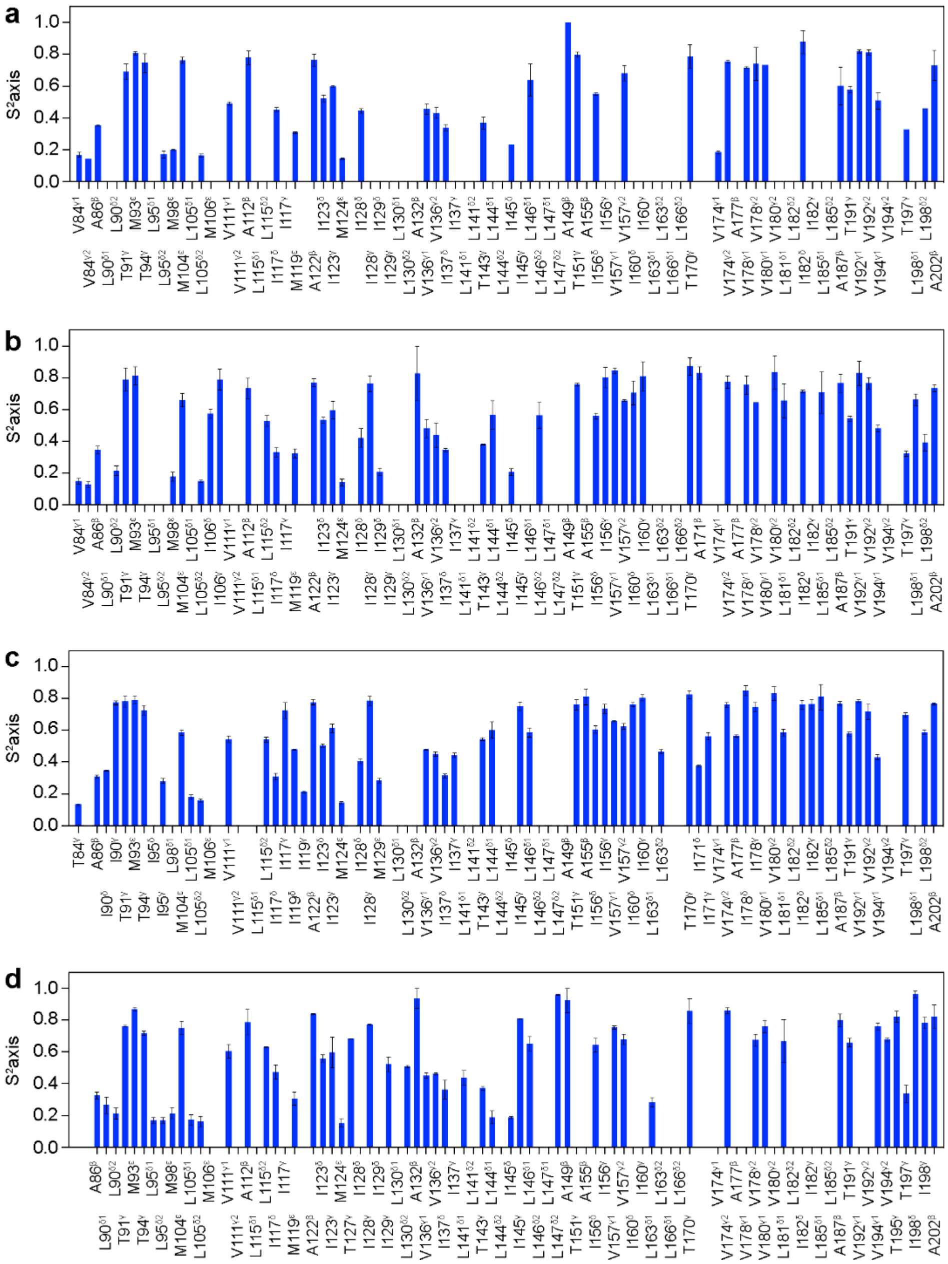

Supplementary Figure 14. NMR order parameters of methyl groups S^2_{axis} in **a** 1918, **b** PR8, **c** Ud, and **d** VN NS1s. Bars and whiskers represent the best fit values and standard error of the fitted parameter, resp ectively. Source data are provided as a Source Data file.

Supplementary Figure 15

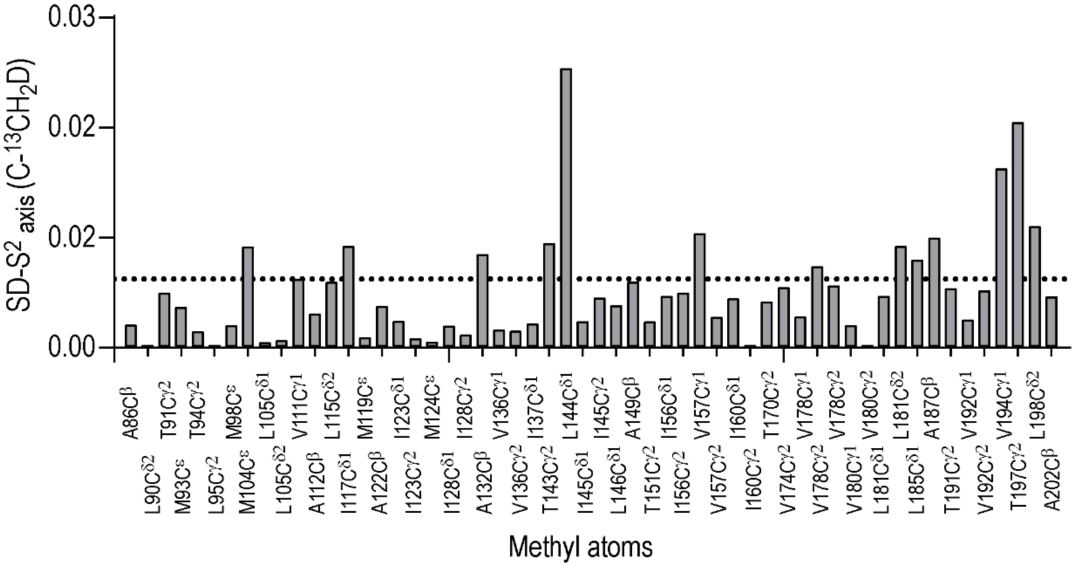

Supplementary Figure 15. Standard deviation of S^2_{axis} values of four NS1 proteins (. Mutated residues were excluded from the calculation. Dotted line represents Q3 of standard deviations. The exact sample size (n) for individual residues varies owing to peak overlap and variations in peak intensities. The sample sizes for individual residues are provided as a Source data file. Source data are provided as a Source Data file.
