# Supplementary material for: Energy landscape reshaped by strain-specific mutations underlies epistasis in NS1 evolution of influenza A virus
Source: Nat Commun. 2022 Oct 1;13:5775. doi: 10.1038/s41467-022-33554-9 (PMC9526705; doi:10.1038/s41467-022-33554-9)
Supplement: Supplementary file 3 — Reporting Summary [file 41467_2022_33554_MOESM3_ESM.pdf]

nature portfolio

Corresponding author(s): Jae-Hyun Cho
Last updated by author(s): Sep 2, 2022

Reporting Summary

Nature Portfolio wishes to improve the reproducibility of the work that we publish. This form provides structure for consistency and transparency in reporting. For further information on Nature Portfolio policies, see our Editorial Policies and the Editorial Policy Checklist.

Statistics

For all statistical analyses, confirm that the following items are present in the figure legend, table legend, main text, or Methods section.

n/a	Confirmed	
☐	☒	The exact sample size (*n*) for each experimental group/condition, given as a discrete number and unit of measurement
☐	☒	A statement on whether measurements were taken from distinct samples or whether the same sample was measured repeatedly
☒	☐	The statistical test(s) used AND whether they are one- or two-sided<br>*Only common tests should be described solely by name; describe more complex techniques in the Methods section.*
☒	☐	A description of all covariates tested
☒	☐	A description of any assumptions or corrections, such as tests of normality and adjustment for multiple comparisons
☐	☒	A full description of the statistical parameters including central tendency (e.g. means) or other basic estimates (e.g. regression coefficient) AND variation (e.g. standard deviation) or associated estimates of uncertainty (e.g. confidence intervals)
☒	☐	For null hypothesis testing, the test statistic (e.g. *F*, *t*, *r*) with confidence intervals, effect sizes, degrees of freedom and *P* value noted<br>*Give P values as exact values whenever suitable.*
☒	☐	For Bayesian analysis, information on the choice of priors and Markov chain Monte Carlo settings
☒	☐	For hierarchical and complex designs, identification of the appropriate level for tests and full reporting of outcomes
☒	☐	Estimates of effect sizes (e.g. Cohen's *d*, Pearson's *r*), indicating how they were calculated

Our web collection on statistics for biologists contains articles on many of the points above.

Software and code

Policy information about availability of computer code

Data collection	X-ray crystal diffraction: R-ASIS IV++ image collector on a Rigaku MicroMax 007HF. NMR: Bruker Topspin 3.2. Fluore BLI: Octet RED biolayer interferometer (Pall ForteBio). Fluorescence spectroscopy: PTI QuantaMaster. ITC: Malvern PEAQ-ITC
Data analysis	BLI data:GraphPad Prism (v9). NMR data: NMRPipe (v10.9), NMRFAM-Sparky (v3.19), Mathematica (v12). X-ray diffraction data analysis: iMosflm (v7.4.0), CCP4 (v7.1.001), Coot, and Phenix package (v1.19.1_4122). ITC: Malvern PEAQ-ITC analysis software (v1.3). Structural analysis: ChimeraX (v1.4) and Pymol (v2.5.2). Protein sequence analysis: Clustal Omega and WebLogo (v3)

For manuscripts utilizing custom algorithms or software that are central to the research but not yet described in published literature, software must be made available to editors and reviewers. We strongly encourage code deposition in a community repository (e.g. GitHub). See the Nature Portfolio guidelines for submitting code & software for further information.

Data

Policy information about availability of data

All manuscripts must include a data availability statement. This statement should provide the following information, where applicable:

- Accession codes, unique identifiers, or web links for publicly available datasets
- A description of any restrictions on data availability
- For clinical datasets or third party data, please ensure that the statement adheres to our policy

All data generated in this study are available within the Article and Supplementary information. The coordinate of VN NS1 in complex with p85β are available in the Protein Data Bank (PDB) under accession code 7RCH (https://www.rcsb.org/structure/7RCH). NMR chemical shifts for backbone atoms are available in the Biological

Magnetic Resonance Bank under accession code 51403 for VN NS1 (https://bmrb.io/data_library/summary/index.php?bmrbId=51403) and 51404 for PR8 NS1 (https://bmrb.io/data_library/summary/index.php?bmrbId=51404). Source data are provided with this paper.

Human research participants

Policy information about studies involving human research participants and Sex and Gender in Research.

Reporting on sex and gender	N/A
Population characteristics	N/A
Recruitment	N/A
Ethics oversight	N/A

Note that full information on the approval of the study protocol must also be provided in the manuscript.

Field-specific reporting

Please select the one below that is the best fit for your research. If you are not sure, read the appropriate sections before making your selection.

[x] Life sciences [ ] Behavioural & social sciences [ ] Ecological, evolutionary & environmental sciences

For a reference copy of the document with all sections, see nature.com/documents/nr-reporting-summary-flat.pdf

Life sciences study design

All studies must disclose on these points even when the disclosure is negative.

Sample size	No statistical methods were used for predetermining the sample size. Experiments were repeated to ensure reproducibility.
Data exclusions	No data exclusion.
Replication	All experiments were repeated at least two or three times. After experimental conditions were optimized, all repeated measurements provided consistent results.
Randomization	No randomization. All prepared samples were used randomly. There was no reason for sample grouping and randomization.
Blinding	No blinding. All experimental data were acquired in the same conditions. All data were examined by multiple people.

Reporting for specific materials, systems and methods

We require information from authors about some types of materials, experimental systems and methods used in many studies. Here, indicate whether each material, system or method listed is relevant to your study. If you are not sure if a list item applies to your research, read the appropriate section before selecting a response.

Materials & experimental systems

n/a	Involved in the study	
[ ]	[x]	Antibodies
[x]	[ ]	Eukaryotic cell lines
[x]	[ ]	Palaeontology and archaeology
[x]	[ ]	Animals and other organisms
[x]	[ ]	Clinical data
[x]	[ ]	Dual use research of concern

Methods

n/a	Involved in the study	
[x]	[ ]	ChIP-seq
[x]	[ ]	Flow cytometry
[x]	[ ]	MRI-based neuroimaging

Antibodies

Antibodies used	Co-IP: Strepavidin magnetic beads (Pierce, Cat. No: 8816) and 6x anti-His antibody (Invitrogen Cat No. MA1-21315-HRP, 1:500 dilution, clone: HIS.H8).
Validation	The following validation was taken from the manufacturer's website.<br>Western blot analysis of 6x-His Epitope Tag was performed by loading various amounts of E. coli lysate containing a multi-epitope

tagged protein per well onto a 4-20% Tris-HCl polyacrylamide gel. Proteins were transferred to a PVDF membrane and blocked with 5% BSA/TBST for at least 1 hour. The membrane was probed with an HRP-conjugated 6x-His Epitope Tag monoclonal antibody (Product # MA1-21315-HRP) at a dilution of 1:1000 overnight at 4°C on a rocking platform and washed in TBS-0.1% Tween-20. Chemiluminescent detection was performed using SuperSignal West Pico (Product # 34080).
